# Zinc-Doped Antibacterial Coating as a Single Approach to Unlock Multifunctional and Highly Resistant Titanium Implant Surfaces

**DOI:** 10.1021/acsami.4c21875

**Published:** 2025-03-18

**Authors:** Samuel S. Malheiros, Maria Helena R. Borges, Elidiane C. Rangel, Carlos A Fortulan, Nilson C. da Cruz, Valentim A. R. Barao, Bruna E. Nagay

**Affiliations:** † Department of Prosthodontics and Periodontology, Piracicaba Dental School, Universidade Estadual de Campinas (UNICAMP), Av. Limeira, 901, Piracicaba, São Paulo 13414-903, Brazil; ‡ Laboratory of Technological Plasmas, Institute of Science and Technology, São Paulo State University (UNESP), Av. Três de Março, 511, Sorocaba, São Paulo 18087-180, Brazil; § Department of Mechanical Engineering, University of São Paulo (USP), Trabalhador São Carlense, 400, São Carlos, São Paulo 13566-590, Brazil

**Keywords:** zinc, dental implant, plasma electrolytic oxidation, biomaterials, bioactive coatings, biofilms, corrosion, proteins

## Abstract

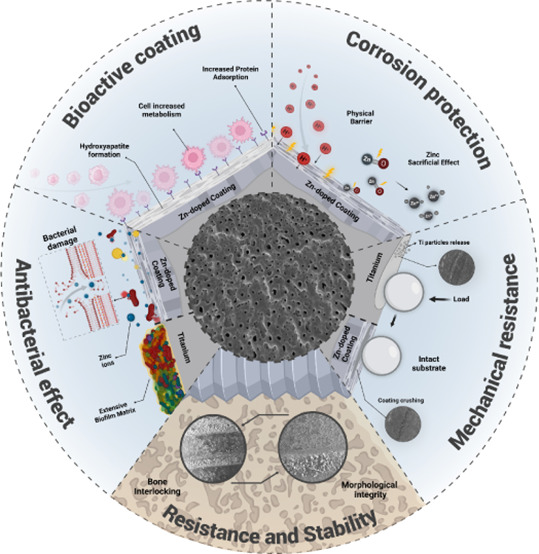

Failures of dental and orthopedic implants due to microbial colonization, corrosion, and insufficient osseointegration remain persistent clinical challenges. Current implant surface coatings often lack the mechanical robustness needed for long-term success. Therefore, this study developed zinc (Zn)-doped coatings on titanium implants via plasma electrolytic oxidation (PEO), achieving 11 at % Zn incorporation primarily as zinc oxide (ZnO). The Zn-doped coatings were primarily composed of zinc, calcium, phosphorus, and oxygen, displaying moderate roughness (∼1 μm), hydrophilic behavior, and high crystallinity with anatase and rutile phases. Tribological tests demonstrated over a 50% reduction in mass loss, while electrochemical tests confirmed significantly enhanced corrosion resistance of Zn-doped coating with higher open circuit potential values, larger Nyquist plot semicircles, and higher impedance values at low frequencies compared to controls (*p* < 0.05). The Zn-doped coatings also showed superior antimicrobial efficacy, reducing *Streptococcus sanguinis* viability, completely inhibiting *Escherichia coli* growth, and reducing biofilm biomass by over 60%, which may be related to the sustained Zn release (∼6 μg/cm^2^) over 7 days. Enhanced bioactivity was evidenced by greater protein adsorption, increased hydroxyapatite formation, and improved preosteoblastic cell metabolism and morphology. *Ex vivo* analyses confirmed coating mechanical stability, without morphological or chemical impairment, during implant insertion and removal from bovine rib bone, with increased implant stability quotient (ISQ) values, indicating benefits in poor bone quality. These findings highlight the significant promise of Zn-doped plasma electrolytic oxidation coatings for advancing dental and orthopedic implant technology, offering enhanced longevity, antimicrobial defense, and improved bioactivity to optimize clinical outcomes.

## 1 Introduction

The global demand for dental implants has been rising significantly, driven in part by their ability to restore function, aesthetics, and to improve the quality of life for millions of patients worldwide.^1^ In the United States alone, dental implant utilization is projected to increase from 5% in 1999 to 23% by 2026.^2^ Mostly implants are produced in titanium (Ti) and titanium alloys due to their good bulk properties.^3^ Despite high survival rates of implants—reported between 90% and 95% over a 10-year period^4^—failures can occur due to various challenges to which implants are subjected during clinical use.^5,6^

Among the critical challenges implants face, the most defiant complications are mechanical, electrochemical, and biological. Mechanical stress begins at the moment of implant insertion, as the processes of drilling and screwing create friction and wear, which can potentially damage the implant surface.^7^ Once placed, implants are subjected to cyclic loading from mastication, which in some cases can induce micromotions at the bone-implant interface, potentially resulting in mechanical fatigue over time.^6^ Simultaneously, implants, especially in oral cavity, are exposed to fluctuating pH levels, temperature variations, and corrosive biological fluids, all of which can compromise their electrochemical stability.^5^ Consequently, the release of Ti ions and particles during wear and corrosion may exacerbate inflammatory responses or accelerate tissue damage.^8^ Additionally, Ti corrosion products have been linked to microbial shifts, facilitating the establishment of dysbiotic biofilms.^9,10^

Biological complications, particularly peri-implant infections driven by biofilm accumulation, are also critical concerns that can lead to implant failure.^11,12^ These infections often arise from the adhesion and accumulation of bacteria on the implant surface.^13^ After colonization, microorganisms start to form biofilms, which are structured communities of microbial cells embedded in a self-produced extracellular polymeric substance matrix, which not only shields bacteria from antimicrobial agents, decontamination techniques and the host immune response but also facilitates the subsequent coaggregation of diverse species.^14^ Also, this matrix allows the creation of anaerobic microenvironments that favor the growth of peri-implant pathogenic bacteria, leading to more aggressive infections.^15^ Notably, the incidence of implant-associated infections varies widely, ranging from 9% to over 30% at patient level.^16^ Biofilm-related infections impose a substantial social and financial burden on patients and healthcare systems, with annual costs exceeding USD 8 billion in the United States alone.^2^ Given their impact, addressing biofilm-related infections remains an urgent priority in biomaterials research.

Despite advances in antimicrobial therapies and decontamination techniques, peri-implant infections remain a significant challenge since there is no accepted gold-standard treatment.^17^ Moreover, the increasing global life expectancy indicates that many implanted patients can present comorbidities such as diabetes, bone and periodontal diseases,^18^ which when uncontrolled may increase infection susceptibility and jeopardize the healing process and, consequently, compromising implant stability.^19^ In response to these challenging scenarios, surface modification techniques have been explored,^20^ with plasma electrolytic oxidation (PEO) being one of the most promissory electrochemical processes since it allows the incorporation of bioactive elements creating coatings with superior properties.^21^ As a low-cost, structurally reliable, and environmentally friendly surface modification method, PEO confers to surfaces superior characteristics in terms of chemical stability, mechanical properties, bioactivity, corrosion and wear resistance compared to other surface treatments.^21^ This is because PEO treatment provides the formation of transient plasma discharges on titanium (and some other valve metals) surface which cause localized heating in the surface facilitating the rapid development of a well-adhered, high crystallinity oxide layer while incorporating bioactive elements.^21^

However, antimicrobial effect by PEO coatings represented a challenge addressed by incorporating antimicrobial agents, with silver being one of the most widely used due to its potent antibacterial properties.^21,22^ However, frequently these ions can induce toxic effects on surrounding tissues, potentially compromising the osseointegration process.^22^ This concern has shifted the focus toward more biocompatible and versatile elements, such as zinc (Zn), an important trace element in the human body. Also, Zn has emerged as an attractive alternative element because it not only can provide coatings with superior mechanical and electrochemical properties by promoting a physical barrier, but it also balances antibacterial efficacy with excellent biocompatibility and bioactivity^23,24^ Its antimicrobial effect is related to the ability of Zn ions to disrupt bacterial cell membranes, inhibit microbial metabolic processes, and reduce biofilm formation.^25−27^ In addition to its antimicrobial properties, Zn plays a crucial role in bone biology by enhancing osteoblast proliferation and differentiation.^28,29^ Furthermore, zinc ability to bind to a significant portion of the human proteome suggests it could enhance protein adsorption on the implant surface, potentially modulating subsequent biological processes.^30^ Therefore, incorporating zinc into PEO coatings presents a promising strategy to develop implant surfaces that are both antimicrobial and conducive to bone healing.

Despite the promising benefits of zinc incorporation,^23,28,31^ previous attempts to integrate zinc into PEO coatings have encountered limitations. First, studies report difficulties in maintaining electrolyte solution stability, often necessitating multiple precursors and complex processing parameters,^32,33^ which complicate the coating production. Additionally, extended treatment times required for a significant zinc incorporation^31^ can adversely affect the overall coating performance, potentially compromising its scalability. Moreover, much of the existing research focuses on surface characterization or PEO parameters effects, without providing a comprehensive evaluation encompassing mechanical, electrochemical, microbiological and biological properties of Zn-doped coatings.^23,28,31^ Yet, the lack of evidence in the literature regarding the resistance of these coatings when submitted to the mechanical stress originated by the implant installation process, limits their clinical translatability. This fragmented approach leaves a significant gap in literature underscoring the necessity for developing simplified fabrication methods, as well as conducting thorough studies to assess the multifunctional capabilities of zinc-doped PEO coatings.

This study explores the development of zinc-doped coatings on titanium implants using a single-step plasma electrolytic oxidation (PEO) process, offering a simplified and efficient approach to creating multifunctional surfaces. After coatings were obtained, physicochemical characterizations of the PEO coatings and control samples were done to establish their structural and compositional properties. The mechanical resistance of the coatings was assessed to evaluate their durability under insertion stresses encountered during implant placement surgeries. The corrosion resistance of the coatings was then investigated, considering the exposure of implants to corrosive bodily fluids. Given the persistent challenge of peri-implant infections, we examined the influence of zinc concentration on the antibacterial performance of the coatings against *Streptococcus sanguinis* and *Escherichia coli*, evaluating biofilm morphology, microbial metabolism, and dry weight. To address the critical need for osseointegration, the bioactivity of the coatings was studied by assessing their ability to adsorb human blood plasma proteins, induce hydroxyapatite formation, and promote preosteoblastic cells viability and adhesion. Finally, the applicability of the coatings was further evaluated through an *ex vivo* test that closely mimicked the natural environment of implant insertion. This included an assessment of surface morphology and chemical composition before and after insertion, as well as implant stability, comparing porous-coated implants to uncoated controls.

## 2 Materials and Methods

### 2.1 Experimental Design

**Figure 1:**
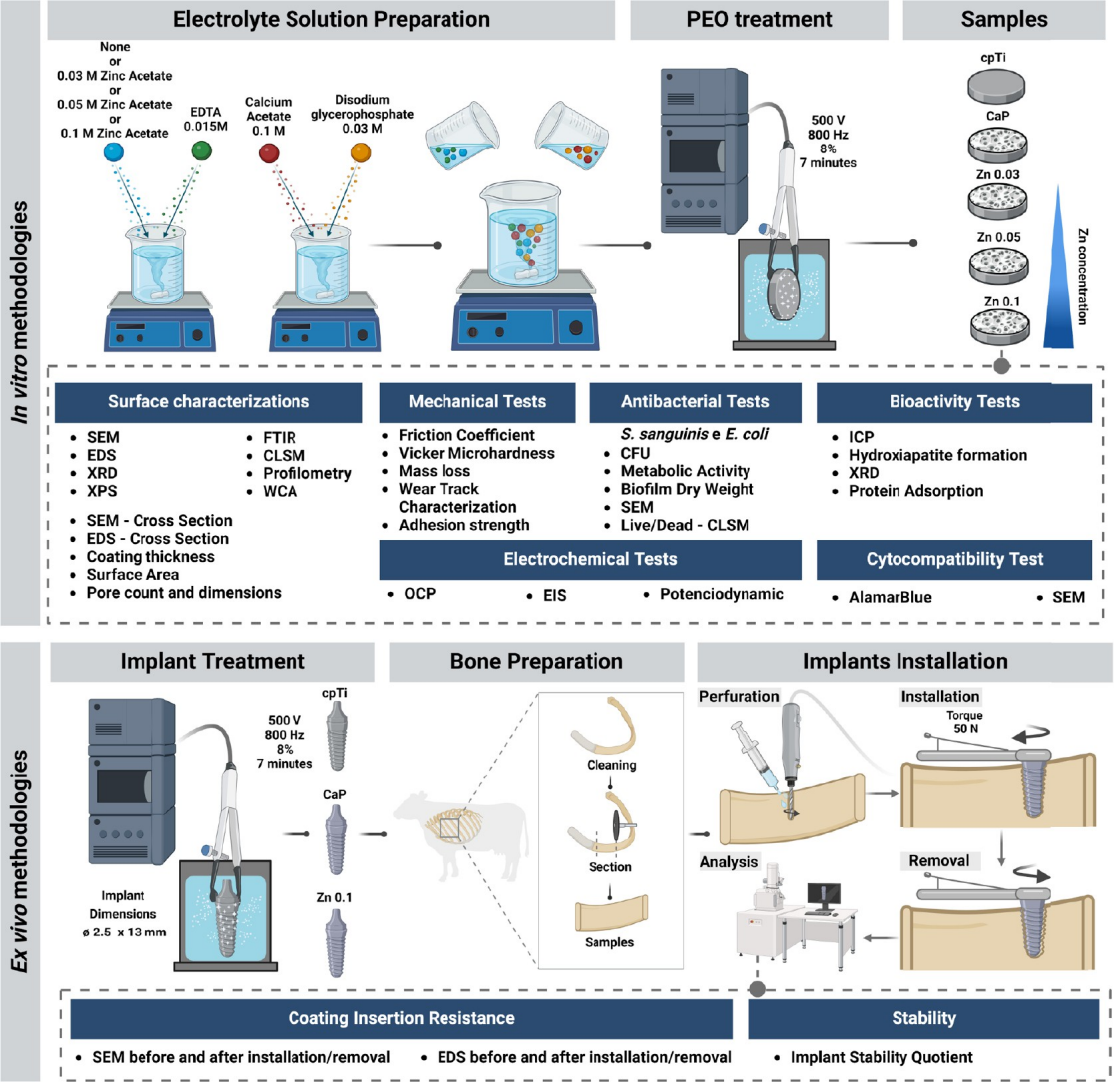
Schematic diagram of the experimental design. SEM = Scanning electron microscopy; EDS = Energy-dispersive X-ray spectrometry; XRD = X-ray diffractometry; CLSM = Confocal laser scanning microscopy; XPS = X-ray photoelectron spectroscopy; FTIR = Fourier transform infrared spectroscopy; WCA = Water contact angle; OCP = Open circuit potential; EIS = Electrochemical impedance spectroscopy; CFU = Colony forming units; ICP = Inductively coupled plasma. Created with BioRender.com (License number: AV27FQ9RWZ).

The experimental design of this combined *in vitro* and *ex vivo* study is illustrated in Figure 1. Commercially pure titanium disks (cpTi; grade II; ø = 10 mm × 2 mm thick, Realum Industria e Comercio de Metais Puros e Ligas Ltd., São Paulo, SP, Brazil) were randomly allocated into five groups for the experimental procedures. Two control groups were established: one comprising polished cpTi disks (cpTi), and the other consisting of PEO-treated disks containing only calcium and phosphorus in the electrolyte solution (CaP). PEO treatment was employed to create zinc bioactive coatings on the experimental groups (Zn0.03; Zn0.05 and Zn0.1, named after each zinc concentration in electrolytic solution). To comprehensively evaluate the properties and bioactivity of the developed coatings, a diverse array of assays was conducted, encompassing physical, chemical, mechanical, tribological, electrochemical, microbiological, and biological analyses. Additionally, the *ex vivo* component of this study assessed the mechanical and chemical resistance of the produced coatings during insertion into bone, simulating dental or orthopedic implant installation, as well as evaluating the coating impact in the implant stability within the bone site. For both cytotoxicity evaluations and the *ex vivo* analyses, only the Zn0.1 experimental group was used as this group consistently demonstrated superior results across all evaluations compared to the other experimental groups.

### 2.2 Surface Preparation

For the *in vitro* experiments, grade II cpTi disks were polished to establish uniform surface conditions for the subsequent treatments. Initially, the disks were polished using #320 and #400 grit SiC abrasive papers (Carbimet 2, Bueheler, Lake Bluff, IL) in an automatic polisher (EcoMet/AutoMet 250 Pro, Buehler). Following the polishing step, the samples were cleaned in an ultrasonic bath with deionized water for 10 min and degreased with 70% propanol for an additional 10 min and then dried using warm air.^34^ For the *ex vivo* study, grade IV titanium implants were provided by DSP Biomedical (DSP Biomedical, Campo Largo, PR, Brazil) with a diameter of 2.5 mm and a length of 13 mm. These implants featured a smooth, polished surface without surface treatments, which was specifically selected to evaluate the effects of PEO treatment on a complex implant geometry. Plasma oxidation treatment was performed using a pulsed direct current (DC) power supply (Plasma Technology Ltd., Kowloon, Honk Kong, China). The cpTi disks/implants served as the anode in the system and were submerged in an electrolyte solution within a stainless-steel tank (cathode) equipped with a cooling system to maintain a temperature of 23 ± 1.5 °C. The electrolyte solution (1 L) was prepared in two steps: first, 0.015 M of Na_2_(EDTA) (C_10_H_14_N_2_O_8_Na_2_·2H_2_O) (99% purity; Dinâmica Ltd., Brazil) was dissolved in 500 mL of distilled water. Subsequently, varying concentrations of 0.03 M, 0.05 and 0.1 M of zinc acetate [Zn(CH_3_CO_2_)_2_·2H_2_O] (99% purity; Synth Ltd., Brazil) were added under agitation at room temperature. The concentrations of Zn0.03, Zn0.05, and Zn0.1, corresponding to the final molarity of zinc in the electrolytic solutions, were selected based on pilot studies to optimize zinc incorporation and evaluate dose-dependent effects. Separately, 0.1 M calcium acetate (C_4_H_8_CaO_4_·H_2_O) (99% purity; Dinâmica Ltd., Brazil) and 0.03 M glycerol phosphate disodium salt hydrate (C_3_H_7_Na_2_O_6_P·H_2_O) (99% purity; Sigma-Aldrich, USA) were dissolved, respectively, in 500 mL of distilled water. Upon complete dissolution of the reagents, both solutions were mixed and used for the PEO process.^32^ The parameters for the PEO treatment were set as follows: a treatment duration of 7 min, with voltage, frequency, and duty cycle of 500 V, 800 Hz and 8%, respectively.^32^ The parameters for the PEO process were determined based on findings from previous literature and refined through pilot studies to ensure optimal coating performance and surface characteristics. Sample labels, electrolyte composition and electrolyte solution conductivity and pH are listed in Table 1. Following the PEO treatment, the samples were thoroughly rinsed with deionized water, air-dried, and stored appropriately.

**Table 1:** Experimental Groups and Electrolyte Composition, Conductivity and pH Used for PEO Treatment

Group	C_4_H_8_CaO_4_ (M)	C_3_H_7_Na_2_O_6_P (M)	Na_2_(EDTA) (M)	C_4_H_6_O_4_Zn (M)	Electrolyte conductivity (mS·cm^–1^)^a^	pH
CaP	0.1	0.03	0.01	-	11.42 ± 0.13	5.84
Zn0.03	0.1	0.03	0.01	0.03	12.75 ± 0.45	5.78
Zn0.05	0.1	0.03	0.01	0.05	13.19 ± 0.47	5.76
Zn0.1	0.1	0.03	0.01	0.1	14.54 ± 0.27	5.74

a Data are expressed as mean standard ± deviation.

### 2.3 Coatings Characterizations

#### 2.3.1 Surface Morphology/Topography

Surface morphology analysis was conducted using Scanning Electron Microscopy (SEM, JEOL JSM-601LA, Peabody, MA, USA) employing electron beans with accelerating voltages of 15.0 kV (*n* = 1/group). Pore area and density were calculated using three SEM images per group at 2,000× magnification using the central area of each image at 50 × 50 μm using ImageJ software (ImageJ 1.53e software; NIH, Bethesda, MD, USA). Cross-sectional images were obtained by SEM following the inclusion of samples (*n* = 3/group) in poly-(methyl methacrylate) (PMMA) resin and subsequent sectioning using a diamond-coated hard-tissue microtome (Leica, Microsystems SP 1600, Nussloch, Germany). Coating thickness was determined by measuring five random areas of the cross-sectioned samples using ImageJ software (ImageJ 1.53e software; NIH, Bethesda, MD, USA). For the surface area and topography analysis, a noncontact three-dimensional Confocal Laser Scanning Microscope (CLSM) (VK-X200 series, Keyence, Osaka, Japan) was utilized to acquire images, which were then processed using the VK-Analyzer software (Keyence v3.3.0.0, Osaka, Japan) (*n* = 3/group).^35^

#### 2.3.2 Zinc Ion’s Release

The release of zinc ions from the coatings was quantified using inductively coupled plasma–optical emission spectrometry (ICP– OES, iCAP 7000, Thermo Scientific). For this, samples (*n* = 3/group) were immersed in 3 mL of phosphate-buffered saline (PBS) at pH 7.4 (Gibco, Life Technologies, The Netherlands) and incubated at 37 °C. Aliquots of the PBS solution were collected at predetermined time intervals (1 h, 4 h, 12 h, 24 h, and 7 days) for analysis. The collected aliquots were diluted appropriately and analyzed to determine the concentration of zinc ions (in ppm).^36^ The Zn ion release (in μg/cm^2^) was calculated by accounting for both the sample surface area (as measured by CLSM) and the volume of the PBS solution.

#### 2.3.3 Roughness

Surface roughness was characterized by measuring the average surface roughness (Ra), root-mean-square (Rq), maximum height of the profile (Rt), and average maximum height of the profile (Rz). A profilometer (Dektak D150; Veeco, Plainview, NY) operating with a cutoff of 0.25 mm at a scan speed of 0.05 mm/s over a duration of 12 s was employed for this purpose.^34^ Three measurements were taken in random areas of each sample (*n* = 5/group).

#### 2.3.4 Wettability

Surface wettability was assessed using the sessile drop method. For this, an automated goniometer (Ramé-Hart Instrument, Co., 0.100–00) measured the contact angle between the sample surfaces (*n* = 3/group) and a droplet of deionized water (10 μL). DROPimage software (Ramé-Hart Instrument Co., Succasunna, NJ) was used to analyze the images and calculate the water contact angle.^34^

#### 2.3.5 Chemical and Crystalline Phase Composition

The chemical composition and atomic proportions were determined using Energy-dispersive X-ray Spectrometry (EDS; JEOL JSM-6010LA, Peabody, MA). Analysis was performed in three random areas of the sample (*n* = 2/group). To analyze the oxidation state of the individual elements, present in each sample (*n* = 1/group), X-ray Photoelectron Spectroscopy (XPS, VSW HA100, Vacuum Science Workshop, Manchester, UK) was employed. The spot size used was 4 × 7 mm, and a step size energy of 0.1 eV was utilized. Reference binding energies were acquired from the National Institute of Standards and Technology XPS Online Database and previous scientific literature. Identification and confirmation of zinc presence in the samples (*n* = 1/group) were conducted using Fourier Transform Infrared Spectroscopy (FTIR, Jasco FTIR 410 spectrometer, Tokyo, Japan). Spectra were acquired in a frequency range from 4000 to 400 cm^–1^, with a resolution of 4 cm^–1^ and an average of 128 scans.^37^ The crystalline phases of the samples (*n* = 1/group) were characterized using X-ray diffraction (XRD; Panalytical, X’Pert3 Powder, Almelo, The Netherlands). The diffractometer utilized Cu-Ka (λ = 1.5418 Å) radiation operating at 45 kV and 40 mA and a continuous speed of 0.02°/second configuration.^38^

### 2.4 Mechanical and Tribological Testing

#### 2.4.1 Microhardness

Vickers microhardness (VHN) of the samples (*n* = 5/group) was determined using an indenter (Shimadzu, HMV-2 Micro Hardness Tester, Shimadzu Corporation, Kyoto, Japan) with an applied load of 0.5 kgf for 15 s.^35^ Three random points on each sample were selected for testing. VHN values were calculated using the following formula:
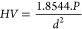
where *P* represents the applied load, and *d* denotes the length of the diagonals of the indentation.

#### 2.4.2 Friction Coefficient, Mass Loss and Wear Track Characterization

A custom-built tribological system (pin-on-disk tribometer) developed at the Faculty of Mechanical Engineering, University of São Paulo, São Carlos, SP, Brazil, was employed to evaluate the wear resistance of the coatings. The counterproof body used was a 5 mm zirconia ball (Tosoh YTX Grinding Media). Tribological tests were conducted with samples (*n* = 3/group) immersed in simulated body fluid (SBF) at 37 °C and pH 7.4. The testing parameters included vertical load of 5 N, track diameter of 7.5 mm, sliding velocity of 0.01 m/second, and sliding duration of 100 s. Further details of the test setup and methodology can be found elsewhere.^35^ Mass loss (μg) was assessed by measuring the disk mass before (baseline) and after the tribological test using a precision balance (AUY-UNIBLOC Analytical Balance, Shimadzu Corporation, Kyoto, Japan). The morphology and calculation of wear scars was characterized according to a previously published protocol^35^ by SEM (JEOL JSM-6010 L A, Peabody, MA, USA). The wear area of samples (*n* = 3/group) was quantified using an optical microscope (VMM-100- BT; Walter UHL, Asslar, Germany) equipped with a digital camera (KC-512NT; Kodo BR Eletrônica Ltd., São Paulo, SP, Brazil) and an analyzer unit (QC 220-HH QuadraCheck 200; Metronics Inc., Bedford, MA, USA). The total surface area of the wear tracks was calculated by measurement of the edges of the discs. Measurements were conducted by a calibrated examiner with an intraclass correlation coefficient (ICC) of 0.848 to ensure measurement accuracy and repeatability.

#### 2.4.3 Adhesion Test

The adhesion strength of the PEO-treated samples was evaluated through a uniaxial tensile test using a Universal Instron mechanical testing system (Instron 4411; Instron Inc., Canton, MA, USA). A clamping fixture was clamped to an 8 mm diameter resin rod securely bonded to the coating surface using cyanoacrylate adhesive (Super Bonder- Loctite, São Paulo, SP, Brazil. The tensile test was conducted by pulling the resin rod at a constant crosshead speed of 1 mm/min until failure of the coating occurred.^34^ After obtaining the quantitative results, the titanium disks were analyzed using SEM to assess the morphological characteristics of the failure sites.

### 2.5 Electrochemical Behavior

As per the method described in prior studies,^35^ the corrosion resistance of the tested samples was evaluated using electrochemical analysis in simulated body fluid (SBF) at 37 °C and pH 7.4. A potentiostat (Interface 1000, Gamry Instruments, Warminster, PA, USA) with a three-electrode cell was employed. The electrochemical techniques included open circuit potential (OCP), electrochemical impedance spectroscopy (EIS), and potentiodynamic polarization, following established protocols. Initially, a cathodic potential of −0.9 V vs SCE was applied for 600 s to standardize the oxide layer. Subsequently, OCP was monitored for 3600 s to determine the free corrosion potential of the material. Next, EIS measurements were conducted over a frequency range of 100 kHz to 5 mHz. Utilizing the Echem Analyst software (Gamry Instruments), EIS data were analyzed applying an appropriate circuit model to each surface. Nyquist, bode (|Z|), and phase angle plots were constructed based on the real (Z”) and imaginary (Z’’) impedance components. Hence, by fitting the real data into an electric circuit, polarization resistance (Rp) and capacitance (Q) parameters were obtained. Potentiodynamic polarization curves were generated by polarizing the samples from −0.8 to 1.8 V. The Tafel extrapolation method was utilized to extract corrosion-related parameters, including corrosion potential (*E*_corr_), corrosion current density (*i*_corr_), Tafel slopes (cathodic - βc, anodic – βa), and corrosion rate. The exposed area (in cm^2^) of each sample (cpTi = 0.89 cm^2^, CaP = 2.18 cm^2^, Zn0.03 = 1.91 cm^2^, Zn0.05 = 1.82 cm^2^, and Zn0.1 = 1.64 cm^2^) was considered for data analysis (*n* = 4/group).

### 2.6 Microbiological Assay

#### 2.6.1 Acquired Pellicle Formation

Prior to microbiological assays, disks were sterilized by UV-light exposure (4 W, λ = 280 nm, Osram Ltd., Berlin, Germany) for 20 min on each side.^35^ To simulate oral cavity conditions and mimic biofilm adhesion on titanium surfaces, an acquired pellicle was formed on all samples using human saliva. Therefore, this study was submitted and approved by the Research Ethics Committee of Piracicaba Dental School, Universidade Estadual de Campinas (UNICAMP) (CAAE: 74390923.7.0000.5418). Freshly stimulated saliva was collected from two healthy volunteers who were selected based on pre-established inclusion and exclusion criteria.^39^ The saliva was centrifuged at 10,000 g for 10 min at 4 °C and filtered using a 0.22 μm membrane filter (K15–1500, Kasvi, São José dos Pinhais, PR, Brazil). Each disk was placed in a 24-well polystyrene cell culture plate, submerged in 1 mL of the filtered saliva, and incubated at 37 °C for 30 min on an orbital shaker (60 rpm) to promote salivary pellicle formation.^40^

#### 2.6.2 Biofilms Growth Conditions

Two monospecies biofilms were cultivated using *Streptococcus sanguinis* (IAL 1832) and *Escherichia coli* (BL21) strains. *S. sanguinis* was chosen due to its role as an initial colonizer of implant surface, favoring the coaggregation of other bacterial species and biofilm formation.^41^ On the other hand, *E. coli* was chosen since it is considered an important species to the perpetuation of implant-related infections.^41^ Also, these species provide valuable insights into the effect of Zn-doped coatings on both Gram-positive and Gram-negative bacteria, which represent different cell structures models. Each bacterial strain was maintained as frozen stocks in 20% glycerol at −80 °C until required. Initially, *S. sanguinis* and *E. coli* were streaked onto Brain Heart Infusion (BHI; Difco Laboratories, Becton, Dickinson and Company, France) and Mueller-Hinton (MH; Becton-Dickinson) agar plates, respectively. The plates were incubated at 37 °C in a 10% CO_2_ atmosphere for 24 h. After incubation, seven colonies of *S. sanguinis* and *E. coli* were transferred into 5 mL of BHI and MH broth medium, respectively, and incubated overnight under the same conditions. Following overnight incubation, 1 mL of each culture was transferred to 9 mL of fresh broth and incubated until reaching the exponential growth phase. Cells were harvested by centrifugation at 6,000 g for 5 min at 4 °C, washed twice with 0.9% NaCl, and resuspended in the corresponding broth medium. The inoculum was then adjusted to an optical density of 1.0 for *S. sanguinis* and 0.1 for *E. coli* at 550 nm, corresponding to approximately 10^7^ cells/mL. Titanium disks with salivary pellicles were placed in 24-well plates containing 900 μL of the appropriate growth medium and 100 μL of the bacterial inoculum. Plates were incubated at 37 °C in a 10% CO_2_ atmosphere for 24 h to promote biofilm formation.^37^

#### 2.6.3 Viability of Microbial Cells

Following 24 h of biofilm growth, the disks (*n* = 6/group) were transferred into cryogenic tubes containing 1 mL of 0.9% NaCl. Biofilms were detached from the disk surfaces by sonication (Branson Sonifier 50, Danbury, CT, USA) with 7 W for 30 s.^35^ A 100 μL aliquot of the sonicated suspension was serially diluted 6-fold, and two 10 μL drops from each dilution were plated on BHI agar for *S. sanguinis* and MH agar for *E. coli*. The plates were incubated at 37 °C with 10% CO_2_ for 48 h. Colony-forming units (CFU) were counted under a stereomicroscope, and results were expressed as log_10_ CFU/mL.^36,37^

#### 2.6.4 Biofilm Dry Weight

To determine biofilm dry weight, which accounts for bacterial biomass, 400 μL of the sonicated suspension were transferred into preweighed microcentrifuge tubes. Three volumes of 100% ethanol were added to the cell suspension, and the tubes were frozen at −80 °C for 20 min. The samples (*n* = 4/group) were then centrifuged at 10,000 g for 10 min at 4 °C. After discarding the supernatant, the tubes were dried under heat and vacuum for 1 h and weighed again.^39^ The dry weight was calculated by subtracting the initial weight from the final weight.

#### 2.6.5 Biofilm Metabolic Activity

Biofilm metabolic activity was assessed using the XTT assay. XTT reagent (Sigma-Aldrich, St. Louis, MO, USA) was dissolved in sterile purified water at a concentration of 0.5 mg/mL and combined with phenazine methosulfate (PMS; Sigma-Aldrich) at 0.32 mg/mL in a 9:1 ratio. A 100 μL aliquot of each sample biofilm suspension (*n* = 4/group) was transferred to a 96-well plate and mixed with 100 μL of the XTT/PMS solution. The plates were incubated in the dark at 37 °C for 30 min to allow the reduction of XTT by metabolically active cells. Colorimetric changes were measured at 492 nm using a spectrophotometer (DU 800 UV–visible Spectrophotometer, Beckman Coulter, Inc.).^42^

#### 2.6.6 Biofilm Structure and Morphology

Biofilm morphology was examined by SEM. Biofilms (*n* = 1/group) were fixed in 2.5% glutaraldehyde for 4 h at room temperature, followed by sequential dehydration in graded ethanol solutions. Samples were air-dried, sputter-coated with gold, and visualized under SEM at an accelerating voltage of 15 kV.^34^ For detailed biofilm three-dimensional analysis, including the visualization of live and dead cells, biofilms were stained using the Live/Dead BacLight bacterial viability kit (Molecular Probes, Eugene, OR, USA), which contains SYTO-9 (for viable cells marking; 480–500 nm) and propidium iodide (for dead cells marking; 490–635 nm). After staining, samples were observed under a confocal microscope (Leica Microsystems CMS, Mannhein, Baden-Württemberg,Germany) at 40 × magnification, using an Ar-ion laser with an excitation wavelength of 488 nm.^43^

### 2.7 Biological and Cytocompatibility Assays

#### 2.7.1 Protein Adsorption

After approval from the Ethics Committee (CAAE: 74390923.7.0000.5418), human blood plasma was used to assess protein adsorption onto the sample surfaces. Initially, disks were incubated with 1 mL of human blood plasma on an orbital shaker at 37 °C and 75 rpm for 2 h. Subsequently, the samples (*n* = 4/group) were gently washed twice with phosphate-buffered saline (PBS) to remove nonadherent proteins and transferred to cryogenic tubes containing 1 mL of PBS. The tubes were then sonicated in a Cup Horn sonicator (5.5-in. cup, Q500, Qsonica, Newtown, CT, USA) at an amplitude of 80% for 60 s to detach the adsorbed proteins.^36^ A 10-fold dilution was performed, and the diluted solution was dispensed into 96-well plates. Protein quantification was conducted using the bicinchoninic acid (BCA) assay. Fresh BCA working reagent (BCA Kit, Sigma-Aldrich, St. Louis, MO, USA) was prepared according to the manufacturer instructions and added to the wells. The plates were incubated for 1 h at 60 °C in the dark. Protein concentration was determined based on a standard curve generated with bovine serum albumin (BSA) standards, and the absorbance was measured at 562 nm using a microplate reader (Multiskan, Thermo Scientific, Vantaa, Finland).^35^

#### 2.7.2 Hydroxyapatite Formation

The ability of the coatings to induce hydroxyapatite formation was assessed by immersing the samples (*n* = 2/group) in SBF solution for 28 days. Each sample was immersed in a volume of 10 mL of SBF per cm^2^ of surface area and incubated under static conditions at 37 ± 1 °C. The SBF solution was prepared according to Kokubo’s protocol to simulate the ionic concentration of human plasma. After the immersion period, the samples were gently rinsed with deionized water and air-dried. SEM images were obtained to observe morphological features indicative of apatite deposition. Hydroxyapatite formation on the sample surfaces was evaluated using EDS and XRD to identify corrosion products and crystalline phases corresponding to hydroxyapatite, respectively.^34^

#### 2.7.3 Preosteoblastic Cell Culture

To assess the cytocompatibility of the surfaces, preosteoblastic MC3T3-E1 cells, derived from mouse calvarial bone, were obtained from a certified cell bank (ATCC CRL-2594; Banco de Células do Rio de Janeiro) and cultured directly on the sample surfaces. The cells were maintained in α-Minimum Essential Medium (α-MEM; Gibco, Life Technologies, USA), supplemented with 100 U/mL penicillin, 100 μg/mL streptomycin, and 10% fetal bovine serum (FBS; Gibco, Life Technologies, Grand Island, NY, USA), under standard cell culture conditions of 5% CO_2_ and 37 °C. Upon reaching approximately 80% confluence, the cells were detached using trypsin-ethylenediaminetetraacetic acid (Gibco) and resuspended in supplemented culture medium for seeding onto the samples. Before cell seeding, each titanium disk was preincubated with 150 μL of FBS for 1 h to simulate the formation of a protein-rich pellicle, mimicking *in vivo* conditions. Then, samples were transferred to a new 48-well polystyrene plate, and MC3T3-E1 cells were seeded into disks surfaces at a density of 1 × 10^4^ cells per well. The plates were maintained for 24 h under controlled conditions to allow for monolayer cell formation, according to the standardized testing method of direct contact described by the International Organization for Standardization (ISO) 10993–5. The culture medium was replaced every other day throughout the experiment to maintain optimal conditions for cell growth and surface interaction.^38,43^

#### 2.7.4 Cell Viability

The effect of the experimental surfaces on the metabolic activity of MC3T3-E1 cells was assessed using the alamarBlue assay. At predetermined time points (1, 3, and 7 days), the culture medium was removed, and 500 μL of fresh medium containing 10% alamarBlue reagent (Invitrogen, Carlsbad, CA, USA) was added to each well. The plates were incubated at 37 °C for 4 h to allow for the reduction of alamarBlue to its fluorescent form by metabolically active cells. Subsequently, 100 μL of the solution from each well were transferred to a 96-well plate for absorbance measurement. The absorbance values were quantified using a microplate reader (Multiskan, Thermo Scientific, Vantaa, Finland) at 570 and 600 nm.^38^ The percentage of cell viability was calculated relative to the cpTi control group at day 1. The cellular morphology on the sample surfaces was analyzed using SEM (JEOL JSM-6010LA, Japan). For sample preparation, cultivated cells were fixed with a 2.5% glutaraldehyde solution and sequentially dehydrated using ethanol solutions of increasing concentrations. Subsequently, the samples underwent critical-point drying (model DCP-1; Denton Vacuum, USA), were coated with a thin layer of gold via sputtering and then imaged to assess morphological characteristics.^38^

### 2.8 *Ex Vivo* Assay

To evaluate the mechanical resistance of the surface coating during insertion into bone, simulating its clinical application in dental and orthopedic implants, an *ex vivo* model was employed. Bovine rib fragments of approximately 10 cm in length and 4 cm in height, with type I bone density—characterized by a thick cortical layer and reduced trabecular bone content—were selected due to their anatomical similarity to human cortical bone and given this characteristic, they could have a greater tendency to cause morphological damage to the surface.^44^ Only fragments displaying the highest degree of homogeneity in morphology and density were included to ensure experimental consistency.^7^ The bone fragments were prepared by removing all soft tissue, including muscle and periosteum, followed by disinfection. Bone drilling was conducted in strict adherence to the implant manufacturer protocols. A drilling system was employed, beginning with a lance drill to create an initial entry point, followed by a 2 mm cylindrical drill, both powered by an electric motor. Drilling was performed at a constant rotational speed of 800 rotations per minute (RPM) under continuous irrigation with sterile 0.9% NaCl solution to maintain bone integrity and prevent thermal damage. Following bone preparation, PEO-treated and untreated implants were inserted into the drilled sites at a controlled rotational speed of 30 rpm, using a surgical motor with torque control set to 50 N/cm. To control intersample variability in bone characteristics, each bone fragment received one implant from each group in a random order, thereby minimizing the impact of individual differences in bone morphology on the outcomes. Upon insertion, the implants were carefully removed using a torque wrench and stored under controlled conditions.

#### 2.8.1 Morphological and Chemical Analysis

To assess the impact of implant insertion into bone on the integrity of the surface coating, implants from each experimental group (*n* = 2/group) were evaluated both before and after insertion. Morphological changes and potential damage to the surface were examined using SEM (JEOL JSM-601LA, Peabody, MA, USA). Prior to SEM and EDS analysis, all implant faces were examined using optical microscopy to identify the most representative surface. SEM analysis was performed using electron beams with an accelerating voltage of 8.0 kV and a spot size of 70 to optimize resolution and image acquisition. Micrographs of the three thirds of the implant were obtained to detect defects or delamination of the coating. The chemical composition and atomic proportions (%) of the coatings were determined via EDS (JEOL JSM-6010LA, Peabody, MA, USA). EDS analysis was conducted in randomly selected regions in each third of each sample, both before and after insertion, to detect any changes in elemental composition that might indicate coating degradation or material transfer. Elemental distribution was further assessed through color mapping to provide a visual representation of the spatial distribution of key elements within the coating and the possible loss of them upon insertion.

#### 2.8.2 Implant Stability Quotient

Implant stability was quantified using the resonance frequency analysis (RFA) method, which measures the implant stability quotient (ISQ), reflecting the stiffness of the bone-implant interface. The ISQ values were recorded using the Penguin RFA system (Integration Diagnostics AB, Göteborg, Sweden) and were presented on a scale ranging from 1 (indicating the lowest stability) to 100 (indicating the highest stability).^45^ For each implant, the ISQ was measured using a commercially available transducer (multipeg) specifically adapted to the implants. The measuring device was positioned perpendicular to the multipeg, ensuring proximity to its uppermost portion without contact. Four measurements were taken per implant (*n* = 3/group), rotating the transducer 90 deg between each measurement to capture stability in different directions. ISQ values were recorded immediately after implant placement.

### 2.9 Statistical Analysis

The sample size for each test was calculated using G*Power software (Heinrich Heine University, Düsseldorf, Germany), ensuring sufficient statistical power based on pilot studies and previously published data. The calculations were performed with an alpha level of 0.05 and a beta of 0.80 to detect significant statistical differences between groups. After data collection, descriptive statistics, including means and standard deviations, were computed. The normality of the data distribution was assessed using the Shapiro-Wilk test, while homoscedasticity was evaluated using Levene’s test to confirm the appropriateness of parametric testing. One-way analysis of variance (ANOVA) was employed to evaluate the effects of surface treatment as the independent variable. In cases where the assumption of homogeneity of variances was violated, Welch’s correction was applied to the ANOVA model. For post hoc multiple comparisons, Tukey’s test was applied to identify statistically significant differences between groups. Statistical analyses were performed using SPSS software (IBM Corp., Armonk, NY, USA). Graphs were generated using GraphPad Prism software (GraphPad Software, San Diego, CA, USA). A significance level of α = 0.05 was consistently applied in all analyses to determine statistical significance.

## 3 Results and Discussion

### 3.1 Coating Morphology, Thickness and Surface Properties

**Figure 2:**
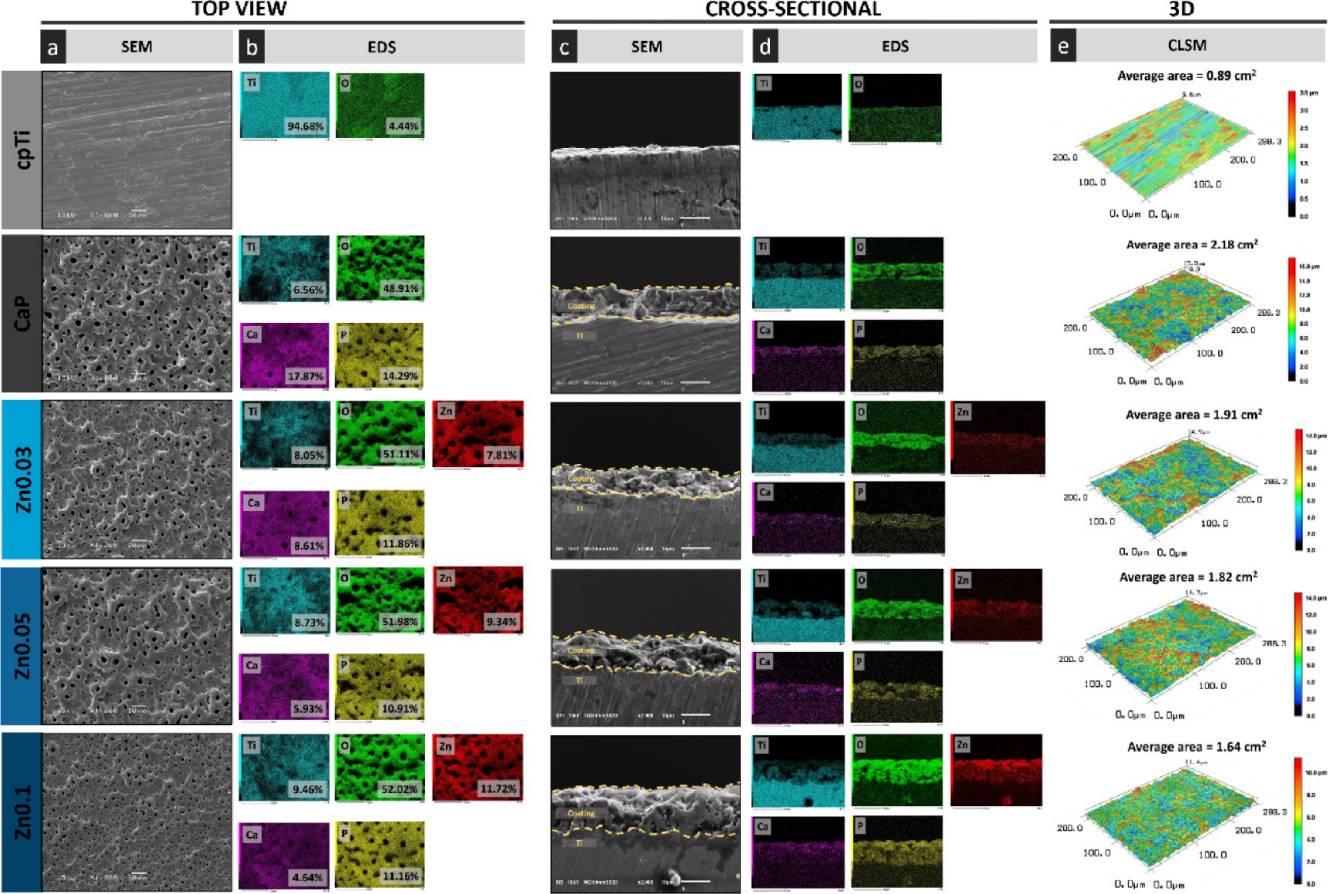
Topographic characterization of control and PEO-treated surfaces. (a) Scanning electron microscopy (SEM) images showing top view surface morphology (scale bar = 10 μm, magnification 1000 × , 15 kV). (b) Energy-dispersive X-ray spectroscopy (EDS) surface maps, highlighting the atomic percentage of individual elements (magnification 2000 × , 10 kV). (c) Cross-sectional SEM analysis of the PEO coatings (scale bar = 10 μm, magnification 2000 × , 10 kV). (d) Cross-sectional EDS maps indicating the spatial distribution of elements across the coating layers (scale bar = 10 μm, magnification 2000 × , 10 kV). (e) Three-dimensional representations of the surface topography obtained via confocal laser scanning microscopy (magnification 50 × ), including the average surface area measurements.

**Figure 3:**
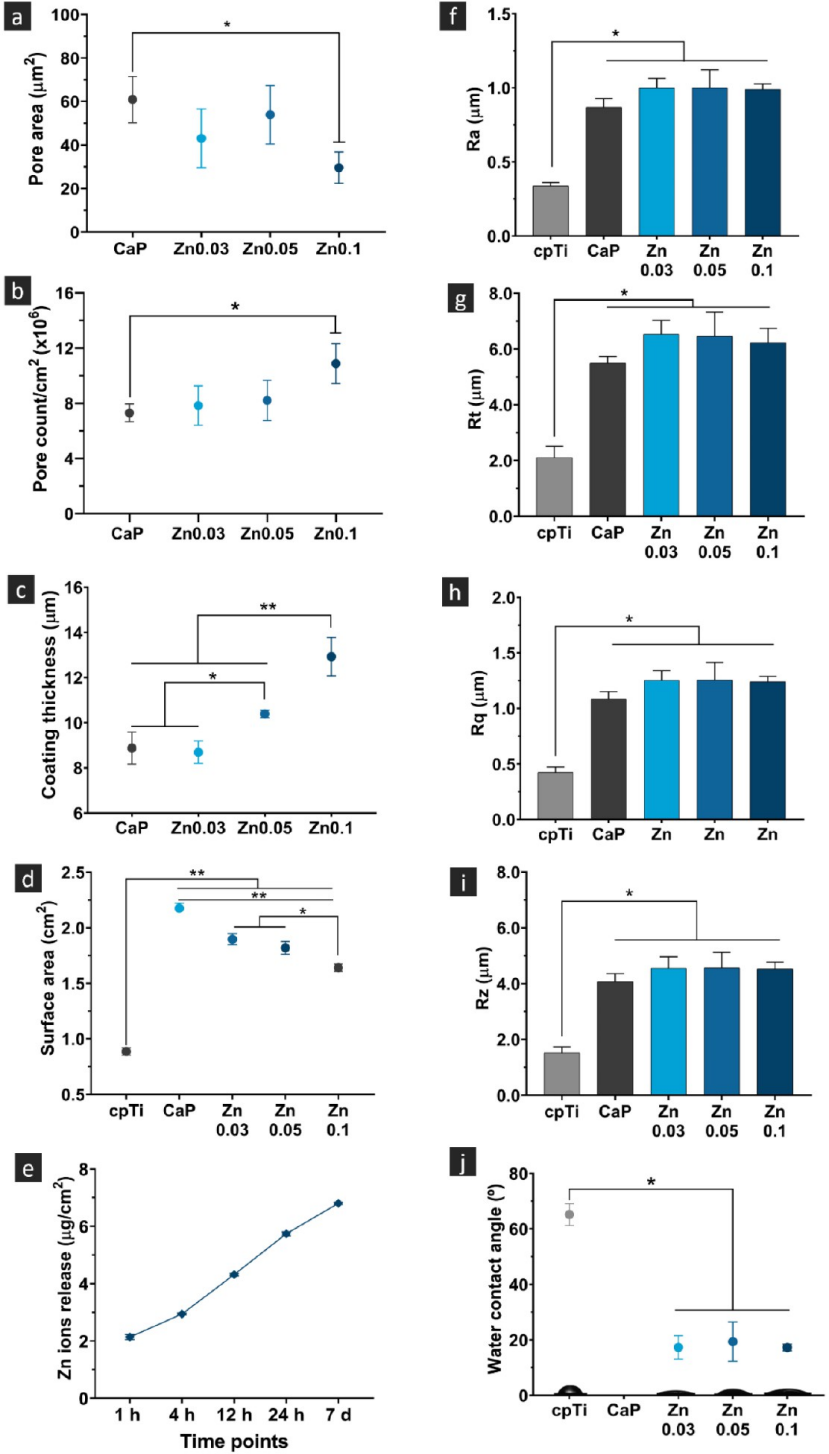
Structural and physicochemical properties characterization of cpTi and PEO-treated samples. (a) Pore area and (b) pore density was determined from SEM micrographs at 2000 × magnification (*n* = 3). (c) Coating thickness of cpTi and PEO samples (*n* = 1, measurements taken at 5 distinct points). (d) Surface area calculated from three-dimensional CLSM images (*n* = 3). (e) Cumulative zinc ions released in PBS over 7 days (*n* = 3). Surface roughness measurements (*n* = 5) for cpTi and PEO groups are represented as (f) Ra (average roughness), (g) Rt (maximum height of the profile), (h) Rq (root-mean-square roughness), and (i) Rz (average maximum height). (j) Water contact angle values (*n* = 5) and representative images of water droplets on control and experimental surfaces. Data are expressed as mean ± standard deviation. Statistically significant differences between groups are indicated by symbols, with * representing *p* < 0.05 and ** representing *p* < 0.001, as determined by Tukey HSD test.

SEM micrographs (Figure 2a) revealed the surface morphology of the examined groups. The cpTi surface displayed longitudinal grooves from the polishing process, while the PEO-treated surfaces exhibited a porous morphology with interconnected pores. All PEO groups showed distinct volcano-like structures, likely caused by high-voltage parameters that expelled molten material through discharge channels, followed by rapid solidification.^21^ Interestingly, the Zn0.1 surface displayed a more uniform appearance, verified by their lower pore area (Figure 3a) and higher pore density (Figure 3b) compared to CaP group (*p* < 0.0415).

Cross-sectional SEM micrographs (Figure 2c) confirmed thick coatings for all PEO-treated groups, with the Zn0.1 group showing the thickest coating at approximately 13 μm, significantly higher than the other groups (*p* < 0.0001; Figure 3c). Additionally, EDS color maps (Figure 2d) demonstrated a uniform distribution of elements within the coating, reflecting the chemical incorporation achieved through the PEO process. This uniformity and increased thickness can be attributed to the higher electroconductivity of the Zn0.1 electrolyte solution (Table 1), which likely facilitated the formation of a gas envelope around the substrate. This envelope increased the energy required to induce numerous low intensity microdischarges, resulting in the formation of microbubbles,^21^ ultimately leading to a higher pore density and reduced pore area on the surface.^36^

Complementing the SEM findings, the three-dimensional images obtained by CLSM (Figure 2e) and laser and optical merged images (see Figure S1) supported the SEM analysis. The CaP, Zn0.03, and Zn0.05 groups showed greater vertical discrepancy compared to Zn0.1, evidenced by more pronounced peaks and valleys, represented by intense orange and blue shades. This resulted in a higher average surface area (Figure 3d) for the CaP, Zn0.03, and Zn0.05 groups compared to Zn0.1 (*p* < 0.0048). Quantifying the surface area of the coating was crucial for assessing its zinc ion release, a key characteristic influencing its functional performance. As illustrated in Figure 3e, the Zn0.1-coating displayed an initial increased zinc ion release within the first 24 h, followed by a slower, sustained release over time. After 1 h, the coating released approximately 2 μg/cm^2^ of zinc, nearly tripling by the 24-h mark. Although the release rate decreased after 24 h, it remained continuous, indicating prolonged zinc availability on the surface. This release pattern suggests potential for antimicrobial activity, essential in preventing bacterial colonization during the early stages postimplantation. Moreover, the continuous release may enhance osseointegration by supporting bone healing over time.

Despite differences in surface area, all PEO-treated samples had similar average surface roughness (Ra) values of approximately 1 μm (*p* > 0.05; Figure 3f), significantly greater than cpTi (*p* < 0.001). Other roughness parameters (Rt, Rq, Rz) followed this trend (Figure 3g–i). The increased roughness in PEO-treated groups is linked to pore and outgrowth formation during the process. Although the optimal roughness for enhancing biological properties is debated, moderate roughness (Ra ∼ 1 μm) has been reported to promote osteoblast proliferation, differentiation, and the expression of alkaline phosphatase, osteocalcin, and VEGF. Moreover, moderately rough surfaces have been associated *in vitro* with a decrease in osteoclast-associated features^46^ while *in vivo*, this type of surface promoted improved osseointegration.^47^

In addition to roughness, surface wettability was also assessed (Figure 3j), as that property plays a crucial role in biological interactions. All PEO-treated samples showed significantly lower water contact angles (WCA) compared to cpTi (*p* < 0.0001). The CaP group displayed superhydrophilic behavior, with water droplets being immediately absorbed by the surface, rendering the WCA measurement impractical and considered as 0°.^43^ This behavior is attributed to the PEO process, where high-energy plasma discharges generate hydroxyl radicals (•OH), increasing surface polarity and hydrophilicity.^36^ Additionally, calcium and phosphorus compounds on the surface may contribute to hydroxyl group availability, further enhancing hydrophilicity through hydrogen bond formation and water spreading.^36^ All zinc-doped groups also exhibited hydrophilic surfaces with similar WCAs, indicating that zinc concentration does not significantly alter the water contact angle. However, variations in Ca^2+^ and PO_4_^3–^ content within the zinc-doped coatings may explain their hydrophilic properties, though less pronounced than the CaP group. Hydrophilic surfaces are advantageous for facilitating protein adsorption and cell-material interactions.^48^ Moreover, they improve affinity with blood factors, thrombin, and blood cells, promoting chemotactic stimuli that induce osteoblast-like cell proliferation.^49^ This enhanced cell-material interaction may accelerate bone-to-metal contact, expediting osseointegration.

### 3.2 Chemical Characterization

Top-view EDS mapping (Figure 2b) revealed that the Zn0.03, Zn0.05, and Zn0.1 coatings were primarily composed of oxygen, calcium, phosphorus, and zinc, uniformly distributed across their surfaces. All PEO-treated samples showed a significant increase in oxygen content, consistent with oxide layer formation during the plasma oxidation process.^21^ Additionally, higher zinc concentrations in the electrolyte solution corresponded to increased zinc incorporation into the coating. Elemental incorporation from the electrolyte solution to PEO coatings involves processes such as electromigration, adsorption, and diffusion.^21^ Zinc ions dissociate from their soluble precursor, zinc acetate, enabling the introduction of cationic Zn^2+^ into the coating primarily through diffusion. However, the degree of zinc incorporation via diffusion is constrained by the concentration gradient, a limitation observed in previous studies.^28,50,51^

To overcome this limitation, we employed two techniques. First, we used EDTA as a chelating agent to form a Zn-EDTA complex,^36^ which keeps zinc soluble and prevents its immediate precipitation with glycerophosphate in the electrolyte solution. This complexation controls zinc ion availability during the PEO process, allowing for the slow, controlled release of zinc ions. Additionally, it promotes the introduction of zinc into the coating not only by diffusion but also by electromigration,^52^ the movement of charged species between the anode (substrate) and cathode under an electric field. Second, to enhance zinc ion flux into the coating via electromigration, PEO parameters were optimized by increasing voltage and frequency. This adjustment strengthened and increased the frequency of microdischarges, facilitating greater zinc incorporation into the coating, as confirmed by EDS (Figure 2b). Notably, higher zinc incorporation was accompanied by a slight reduction in phosphorus content and a significant decrease in calcium concentration within the coating. This phenomenon may result from repulsive interactions between calcium and zinc cations during the chemical incorporation process.^52^

**Figure 4:**
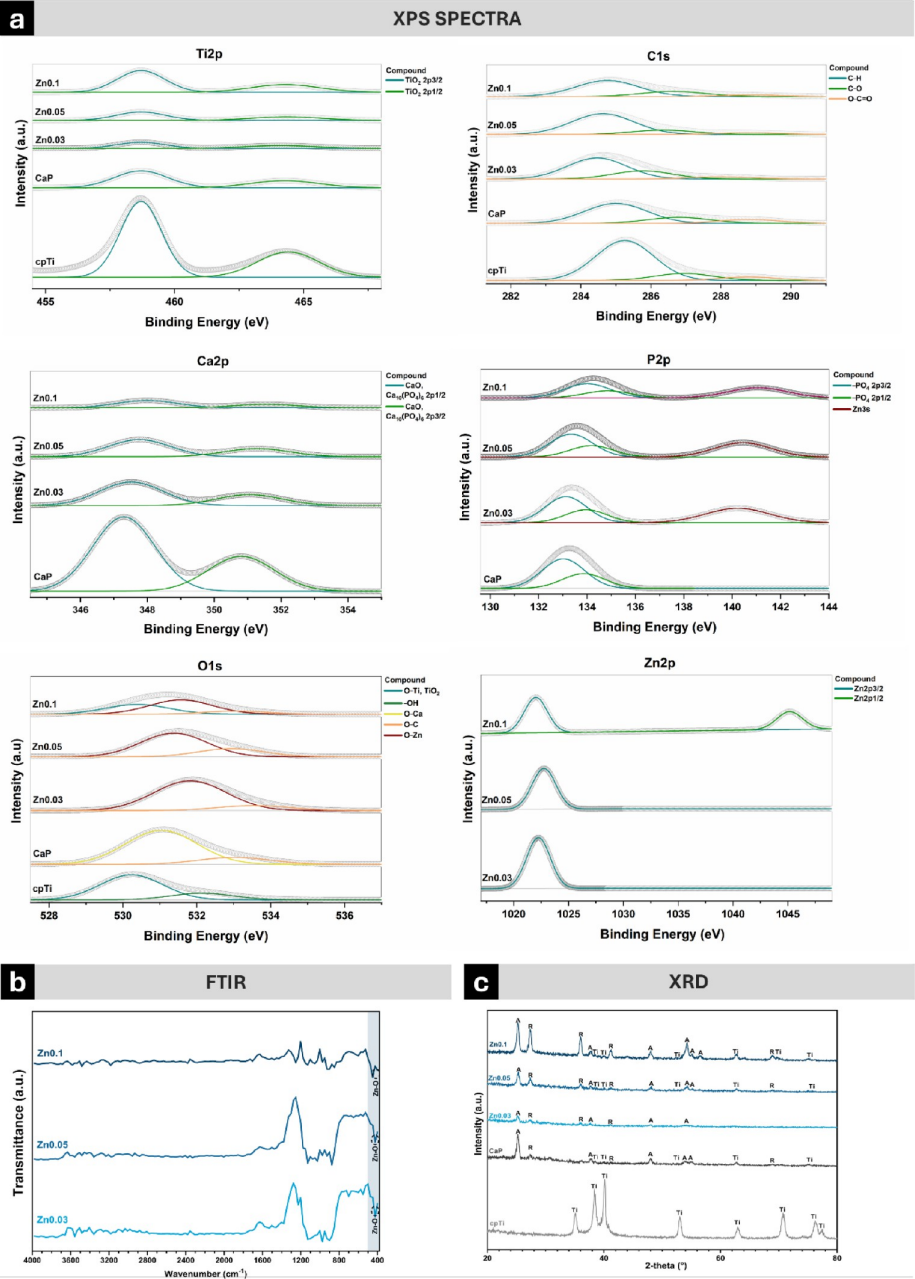
Chemical and structural characterization of control and PEO-treated samples. (a) X-ray photoelectron spectroscopy (XPS) spectra for control and PEO-treated groups (*n* = 1). (b) Fourier-transform infrared spectroscopy (FTIR) spectra (*n* = 1) of cpTi and PEO-treated samples, with dashed circles highlighting chemical bonds characteristic of zinc oxide. (c) X-ray diffraction (XRD) pattern (*n* = 1) of cpTi and PEO-treated samples, indicating the presence of Ti = titanium, A = anatase, and *R* = rutile phases.

Transitioning from elemental composition to chemical states, XPS analysis provided detailed insights into the chemical states of elements and confirmed the successful incorporation of zinc into the coatings. Figure 4a shows the deconvolution of Ti 2p, C 1s, O 1s, Ca 2p, P 2p, and Zn 2p high-resolution spectra for each group. The Ti 2p spectra confirmed the presence of TiO_2_ in the coatings, while the O 1s spectra revealed zinc–oxygen bonds (∼531.5 eV) in the Zn-doped groups, indicative of zinc oxide formation.^28^ The Ca 2p spectra indicated the presence of a calcium oxide layer in all treated groups, but with reduced intensity in Zn-doped coatings, consistent with diminished calcium incorporation as shown by EDS analysis. The P 2p spectra revealed peaks corresponding to phosphate compounds.^28^ Interestingly, the detection of Zn 3s spectra (∼141 eV) in the Zn-doped groups suggests the formation of zinc phosphate within the coatings, achieved through the interaction between dissociated Zn^2+^ ions and PO_4_^3–^ molecules, both formed during the PEO treatment.

The XPS spectra of zinc-doped groups revealed two distinct peaks in the Zn 2p region at ∼1022 eV and ∼1045 eV, corresponding to the Zn 2p3/2 and Zn 2p1/2 states, respectively. These peaks result from spin–orbit coupling, which splits the Zn 2p core level into two components. The Zn 2p3/2 peak at lower binding energy (∼1022 eV) is more intense, primarily indicating the presence of zinc in the Zn^2+^ oxidation state, commonly associated with compounds like zinc oxide (ZnO) and zinc hydroxide (Zn(OH)_2_). The Zn 2p1/2 peak at higher binding energy (∼1045 eV), less intense due to its spin–orbit configuration, was detected exclusively in the Zn0.1 group, suggesting that increased zinc content facilitated clearer detection (Figure S2). The strong Zn 2p3/2 peak across all Zn-doped groups suggests the formation of zinc compounds, while the presence of the Zn 2p1/2 peak at higher zinc concentrations (such as in Zn0.1) underscores the significant amount of zinc incorporated into the coating in both oxide and hydroxide forms.^28,51^

It is proposed that, during the PEO treatment, zinc oxide primarily forms through the reaction of free Zn^2+^ ions with oxygen species generated by the plasma microdischarges.^53^ These oxygen species, such as O^2–^ ions and oxygen radicals, are produced as the electrolyte and water molecules decompose under the high-energy conditions. The Zn^2+^ ions in the electrolyte react with these oxygen species to form ZnO through the reaction as shown in equation (eq 1):^53^
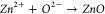
1

This ionic bond formation is supported by the Zn–O peaks detected in the O 1s spectra (∼531.5 eV), which are characteristic of metal oxide formation, indicating that zinc oxide is a significant component of the surface layer. Additionally, the introduction of EDTA into the electrolyte creates a chelating effect, stabilizing Zn^2+^ ions by forming a Zn-EDTA complex. Under the high-temperature conditions of the PEO process, this complex dissociates, releasing Zn^2+^ ions to react with hydroxyl ions (OH^–^) at the surface to form zinc hydroxide (Zn(OH)_2_) (eq 2):^54^
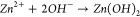
2

As the PEO process continues, the local temperature from the plasma microdischarges increases, causing thermal dehydration of the Zn(OH)_2_ also resulting in the formations of ZnO (eq 3):^53^
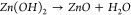
3

In addition to the XPS results, the FTIR analysis further corroborated the presence of zinc in the coatings. As verified in Figure 4b, the absorption peaks near to 450 cm^–1^ can be ascribed to the Zn–O bond as previously verified in the literature,^54,55^ which is also in agreement with the previous finds of XPS analysis. As previously established, chemical composition can significantly affect crystalline phases within the coating,^34^ which was analyzed by XRD.

The XRD diffractograms (Figure 4c) showed peaks of Ti for all surfaces.^34^ PEO-treated surfaces exhibited a reduction in the diffraction peak of the α phase of Ti and presented a mixture of TiO_2_ crystalline phases, with peaks corresponding to anatase and rutile,^36^ indicating that the addition of zinc into the electrolyte solution did not disrupt or create new crystalline phases. Notably, the Zn0.1 group produced higher counts for rutile (26° and 36°) and anatase (25° and 54°) peaks. During PEO treatment, high temperatures within the discharge channels caused melting of the substrate followed by crystallization, promoting oxide layer formation.^21^ Initially, the thin coating allowed an increased reaction rate between Ti and O, leading to the formation of a high amount of titanium oxide, mostly anatase. As the coating thickness increased, the required electrical field intensity was enhanced, producing stronger microdischarges capable of generating the rutile phase on the surface.^21^ Although ZnO typically crystallizes in a wurtzite structure,^54^ no distinct peaks corresponding to zinc oxide (ZnO) were detected in our XRD analysis. This absence suggests that ZnO may not have formed in a crystalline phase detectable by XRD. Possible explanations include the ZnO amount being below the detection limit, formation in an amorphous state that does not produce sharp diffraction peaks, or overshadowing by strong diffraction signals from TiO_2_ phases like anatase and rutile, making ZnO peaks difficult to detect.

According to a recent review, electrical parameters and electrolyte composition can affect the crystalline structure of PEO coatings.^21^ However, Du et al. (2018)^32^ showed that altering the voltage in PEO with zinc did not influence the crystalline phase. It is possible that the higher zinc content in this study, which promoted higher electroconductivity, contributed to this behavior.^32^ Although it was previously reported that the addition of EDTA to the electrolyte solution can jeopardize the thermodynamic process of crystalline phase formation, this was not the case here. We identified several diffraction peaks, especially for the rutile phase that is more favored at high temperatures, indicating that EDTA did not adversely affect the crystalline structure formation. The formation of both anatase and rutile crystalline phases is beneficial for dental implants, as their presence produces bioactive surfaces with superior mechanical and electrochemical properties.^35^

### 3.3 Wear Resistance and Corrosion Protection

**Figure 5:**
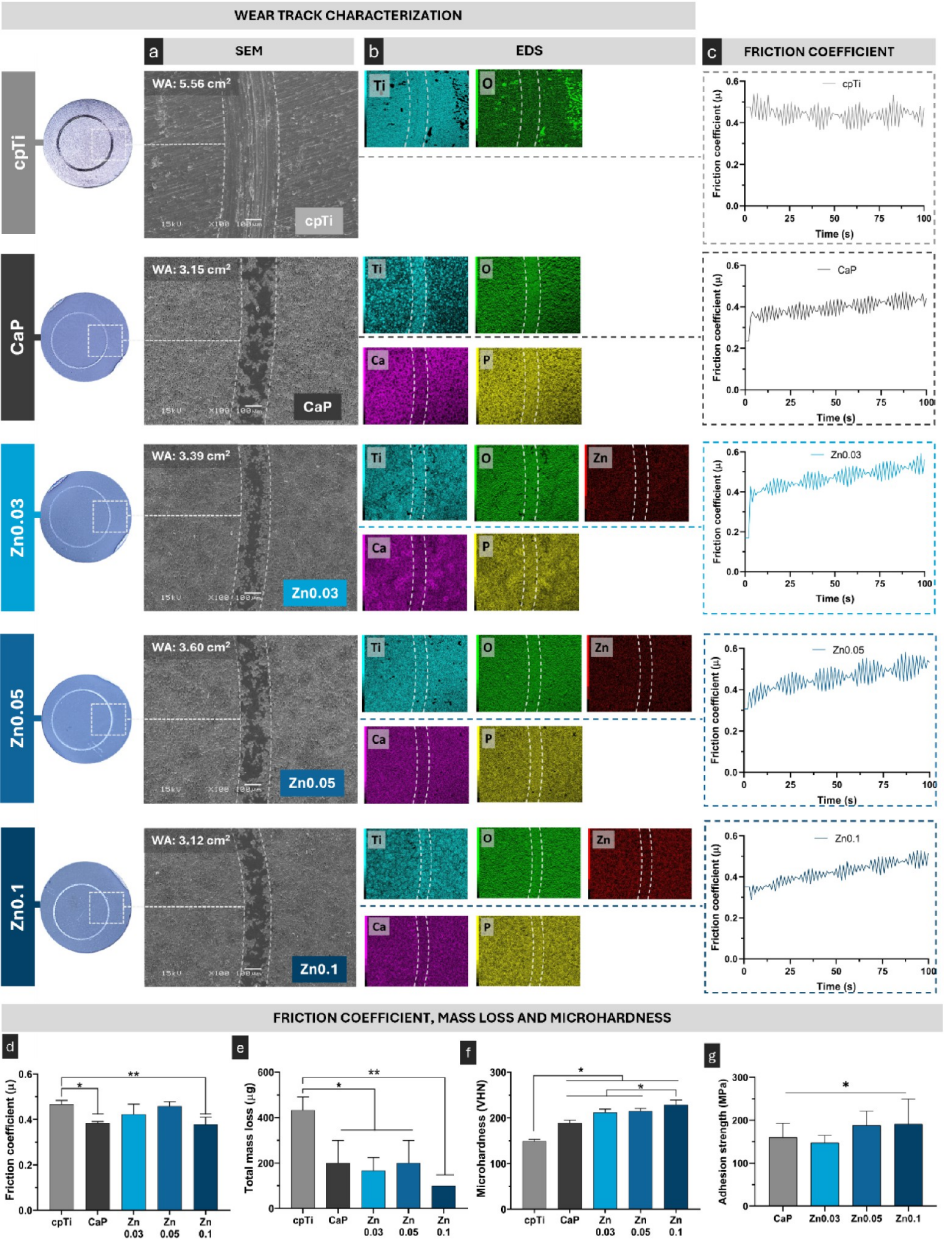
Tribological properties of control and experimental groups. Photographs of disk surfaces post-tribological testing are shown on the left. (a) Scanning electron microscopy (SEM) images (100 × magnification, 15 kV) displaying the wear area after testing, where WA represents the wear area in cm^2^. (b) EDS color maps illustrating the distribution and preservation of the coating and constituent elements, even after mechanical stress. (c) Friction coefficient behavior during tribological testing, and (d) the average sliding friction coefficient (*n* = 3). (e) Total mass loss (mg) of the samples following tribological wear (*n* = 3). (f) Vickers microhardness evaluation of the samples (*n* = 5). (g) Adhesion strength of coatings (*n* = 3). Data are presented as mean ± standard deviation. Statistically significant differences between groups are indicated by symbols, with * denoting *p* < 0.05 and ** denoting *p* < 0.001, based on Tukey HSD test.

A common drawback of titanium coatings is their poor mechanical properties, even more taking into consideration the process of surgical implantation, that can lead to substantial damage to the coating surface.^20^ Therefore, tribological tests were conducted to assess the coating resistance under mechanical stress. Herein, the PEO-developed coating presented excellent mechanical and tribological property (Figure 5). After the test, the wear track characterization (Figure 5a) revealed a narrow wear track for the PEO-treated groups, especially for the Zn0.1, resulting in a lower wear track area (3.12 mm^2^). Yet, the wear tracks not only present different widths, but also different morphologies. In the cpTi group, the wear track appears more evident, with the formation of edges and a central depression that is directly related to the spherical shape of the zirconium ball. On the contrary, groups treated with PEO showed a poorly demarcated track characterized by surface crushing instead of removal. The hypothesis of surface crushing was confirmed by EDS mapping (Figure 5b) that showed that the produced surface treatment was not removed from the surface during the friction, since the incorporated elements such as Ca, P and Zn were still present on the surface and the titanium signals from substrate are not more intense.

Regarding friction coefficients, CaP and Zn0.1 groups presented lower values of friction coefficient compared to the cpTi control group (*p* < 0.032; Figure 5d). The fluctuations in the friction coefficient presented in all the groups are directly related to the accumulation and buildup of particles during the sliding process forming a third body^35^ (Figure 5c) which can justify the increase in the friction coefficient throughout the process. These indications of superior mechanical resistance were further confirmed by the evaluation of mass loss, which was significantly reduced in the PEO-treated groups (*p* < 0.019), and even more for the Zn0.1 group (*p* < 0.002; Figure 5e) compared to cpTi. These results can be related to the larger values of surface microhardness. All PEO-treated samples presented microhardness higher than cpTi (*p* < 0.0001; Figure 5f), probably due to the increase in the oxides into the coating and the presence of the rutile crystalline phase.^35^ All the Zn-treated groups, especially Zn0.1, presented higher microhardness compared to control groups (*p* < 0.03) which can be explained by the large proportion (stronger diffraction peaks) of rutile phase, which has already been proved to enhance surface hardness.^34,38^

The results of the adhesion strength test demonstrated that the coating exhibits high adhesion to the substrate, with force values exceeding 150 MPa (Figure 5g). However, it is important to clarify that this value reflects the cohesive strength of the cyanoacrylate layer rather than an absolute measure of the coating’s adhesion. Despite this, SEM micrographs (Figure S3) confirm the integrity of the PEO coatings after testing, with no evidence of delamination, and cyanoacrylate remnants observed on the surface further support that failure occurred within the adhesive rather than at the coating-substrate interface. This outcome aligns with the fundamental nature of the PEO process, which differs from additive surface modifications techniques where a distinct layer is deposited onto the substrate. Instead, PEO is a surface modification technique that induces localized melting and oxidation of the material, leading to the *in situ* formation of an integrated oxide layer.^21^ As a result, the coating is not merely attached to the substrate but is chemically and mechanically integrated with it, inherently enhancing adhesion. A well-adhered coating is also crucial for long-term corrosion resistance, as delamination or poor interfacial bonding can create pathways for electrolyte infiltration, accelerating substrate degradation.^34^ The excellent tribological performance further substantiates these findings. If the coating were poorly bonded to the substrate, it would have detached under tribological stress, which did not occur. Similarly, in the *ex vivo* tests, the coatings withstood insertion and removal forces without signs of delamination or structural compromise. These results collectively highlight the robustness of the PEO coating, not only in terms of mechanical integrity but also in its capacity to serve as a long-lasting barrier against corrosion.

Those are important data, because dental implants are subjected to the mechanical forces not only during the implant insertion process^7^ but, most important, during all their life in function where is submitted to the physiological forces that can further affect the bone-implant stability.^6^ Also, mechanical stress can lead to implant surface degradation. From a clinical perspective, wear debris can trigger complex inflammatory responses on peri-implant surrounding.^10^ Therefore, the reduced mass loss indicates lower wear debris releasing after mechanical stress pointing to a superior structural integrity of the experimental coatings, which can contribute to homeostasis maintenance.

**Figure 6:**
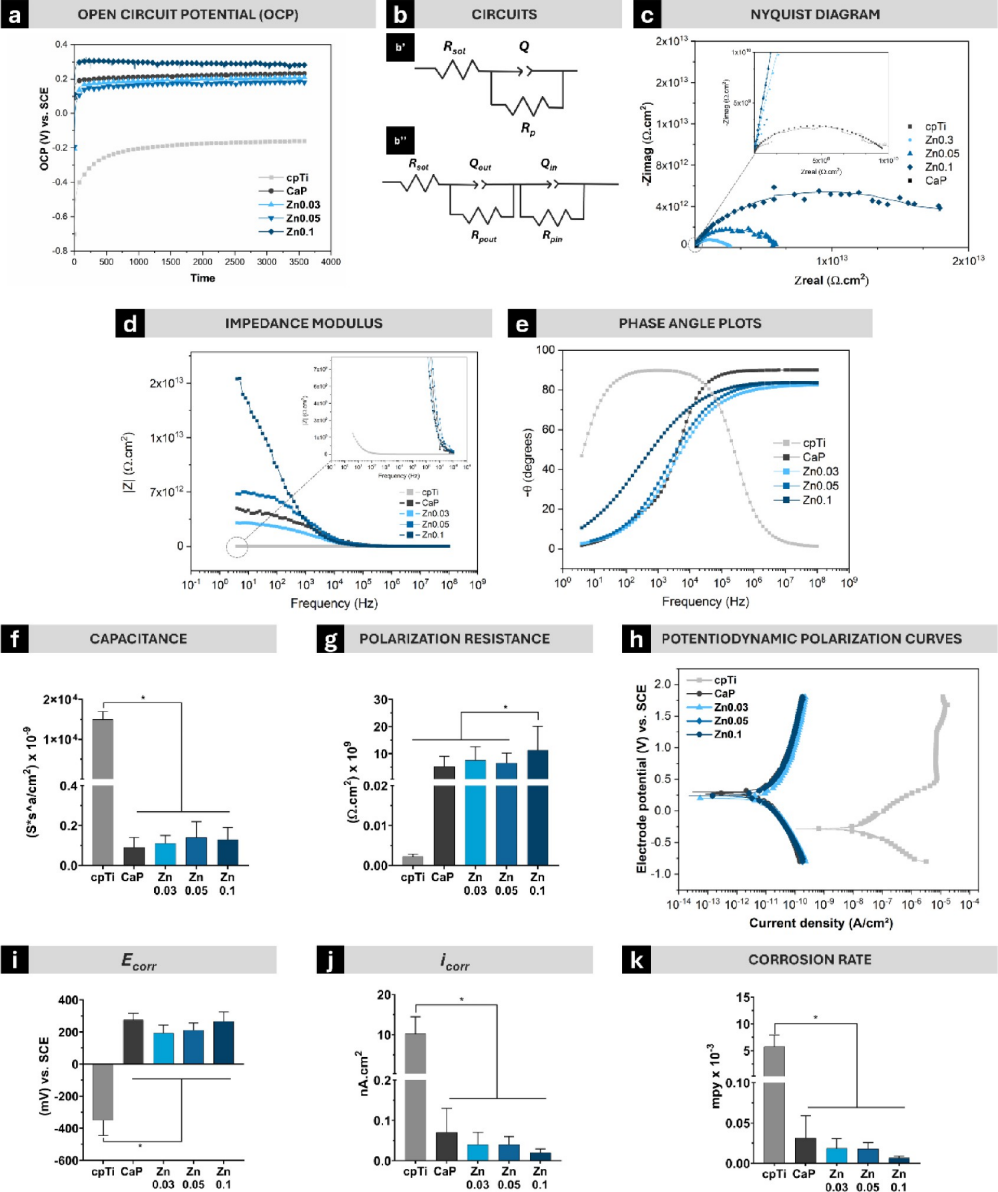
Corrosion evaluation of control and PEO-treated experimental groups. (a) Representative curve showing the evolution of the open circuit potential (OCP) over 3600 s (in V vs SCE—saturated calomel electrode). (b) Equivalent electrical circuit model used for electrochemical impedance spectroscopy (EIS) data, where Rsol represents electrolyte resistance, Rp denotes polarization resistance, and Q is the constant phase element. (c) Nyquist plots, (d) impedance modulus, and (e) phase angles from EIS measurements. Electrical parameters such as (f) polarization resistance and (g) capacitance are derived from EIS data. (h) Potentiodynamic polarization curves (in V vs SCE). (i) Corrosion potential (*E*_corr_), (j) current density of passivation (*i*_corr_), and (k) corrosion rate values. Data are presented as mean ± standard deviation. Statistically significant differences between groups are indicated by symbols, with * representing *p* < 0.05 according to the Tukey HSD test.

The enhanced electrochemical performance of the PEO-treated surfaces was further demonstrated by the open circuit potential (OCP), electrochemical impedance spectroscopy (EIS), and potentiodynamic polarization tests. The electrical and corrosion parameters are summarized in Table 2. OCP measurements revealed significantly higher values for all PEO-treated groups (189.85–280.58 mV vs SCE) compared to cpTi (−160.78 mV vs SCE) (Figure 6a), indicating greater electrochemical stability and lower susceptibility to corrosion.^34^ The equivalent electrical circuits used to simulate the electrochemical behavior of the surfaces are illustrated in Figure 6b. For machined titanium surfaces, a simple circuit comprising solution resistance (Rsol), polarization resistance (Rp), the resistance at the titanium-electrolyte interface and a constant phase element (Q) was applied (Figure 6b’). For PEO-treated surfaces, a more complex model was needed to represent the dual electrochemical interfaces. Two sets of parallel components—*R*_pout_/*Q*_out_ and *R*_pin_/*Q*_in_— were included, representing the polarization resistance and a constant phase element of the porous outer and dense inner oxide layers, respectively (Figure 6b’’).^34^ The suitable agreement between experimental and simulated EIS data, indicated by chi-squared values on the order of 10^–3^ to 10^–4^, demonstrates the robustness fit of the model.

**Table 2:** Mean and Standard Deviations of Electrical and Corrosion Parameters for PEO-Coated Samples^a^

Groups	*R*_pin_ (GΩ.cm^2^)	*R*_pout_ (GΩ.cm^2^)	*R*_tot_ (GΩ.cm^2^)	*Q*_in_ (nΩ.cm^2^)	*Q*_out_ (nΩ.cm^2^)	*Q*_tot_ (nΩ.cm^2^)	η	*X*^2^× 10^–3^	*E*_corr_ (mV) vs SCE	*i*_corr_ (nA.cm^2^)	βa (V dec^–1^)	-βc (V dec-1)	Corrosion rate (mpy)
cpTi	2.22 × 10^–3^ (5.99 × 10^–4^)	-	2.22 × 10^–3^ (5.99 × 10^–4^)^a^	1.50 × 10^4^ (1.98 × 10^3^)	-	1.50 × 10^4^ (1.98 × 10^3^)^a^	0,91 (0.01)	2.89 × 10^4^ (1.36 × 10^4^)	–350 (94.95)^a^	10.2 (4.25)^a^	2.04 (3.31)	0.19 (0.03)	5.75 × 10^–3^ (2.15 × 10^–3^)^a^
CaP	5.15 (3.44)	0.16 (0.26)	5.31 (3.7)^a^	0.08 (0.04)	0.01 (0.01)	0.09 (0.05)^b^	0.72 (0.34)	2.78 × 10^–3^ (3.60 × 10^–3^)	276 (38.97)^b^	0.07 (0.06)^b^	1.40 (1.17)	2.22 (1.93)	3.14 × 10^–5^ (2.77 × 10^–5^)^b^
Zn0.03	7.53 (4.84)	0.10 (0.10)	7.63 (4.95)^a^	0.09 (0.04)	0.02 (0.01)	0.11 (0.04)^b^	0.68 (0.35)	8.58 × 10^–4^ (1.06 × 10^–3^)	192.75 (49.97)^b^	0.04 (0.03)^b^	1.82 (1.28)	1.17 (0.56)	1.86 × 10^–5^ (1.22 × 10^–5^)^b^
Zn0.05	6.44 (3.38)	0.16 (0.27)	6.60 (3.65)^a^	0.11 (0.06)	0.02 (0.01)	0.14 (0.08)^b^	0.70 (0.33)	2.42 × 10^–4^ (1.56 × 10^–4^)	210.5 (45.49)^b^	0.04 (0.02)^b^	1.43 (1.23)	1.51 (1.02)	1.81 × 10^–5^ (7.77 × 10^–6^)^b^
Zn0.1	11.1 (8.55)	0.19 (0.21)	11.24 (8.76)^b^	0.10 (0.05)	0.03 (0.01)	0.13 (0.06)^b^	0.73 (0.30)	1.14 × 10^–3^ (1.43 × 10^–3^)	265 (59.75)^b^	0.02 (0.01)^b^	1.30 (1.40)	0.63 (0.17)	6.96 × 10^–6^ (1.82 × 10^–6^)^b^

a *R*_pin_: Inner polarization resistance;*R*_pout_: Outer polarization resistance; *R*_tot_: Total polarization resistance; *Q*_in_: Inner capacitance; *Q*_out_: Outer capacitance; *Q*_tot_: Total capacitance; η: Efficiency of the coating; *X*^2^ × 10^–3^: Chi-squared value multiplied by 10^–3^, representing the fitting accuracy of the data; *E*_corr_: Corrosion potential; *i*_corr_: Corrosion current density; βA: Anodic Tafel slope; -βc: Cathodic Tafel slope. Means followed by different lowercase letters (a, b) within the same column indicate statistically significant difference among the groups.

The Nyquist plots (Figure 6c) and impedance modulus |Z| values (Figure 6d) further confirmed these findings. At lowest frequencies, the impedance modulus values for the cpTi group were approximately 1 × 10^9^ Ω·cm^2^. In contrast, significantly higher values were observed for the PEO-coated groups, with CaP, Zn0.03, and Zn0.05 reaching approximately 7 × 10^12^ Ω·cm^2^, while the Zn0.1 group exhibited the highest value of around 2 × 10^13^ Ω·cm^2^. This higher impedance of Zn0.1 coating, coupled with higher phase angles at low frequencies (Figure 6e), indicates the formation of a dense, protective oxide layer with superior barrier properties.^35^ At higher frequencies (around 10^6^ Hz), the cpTi group exhibited a phase angle (θ) of approximately −20°, indicating a more resistive behavior and limited capacitive response. In contrast, the PEO-treated groups displayed phase angles close to −90°, reflecting a predominantly capacitive behavior. This suggests that the PEO coatings act as effective barriers, providing superior protection by isolating the substrate from the corrosive environment.

The charge transfer resistance, represented as *R*_pin_ in this study, reflects the resistance to electron transfer at the metal–electrolyte interface and is a key parameter for evaluating corrosion resistance. For the cpTi group, *R*_pin_ was measured as 2.22 × 10^–3^ GΩ·cm^2^, which is significantly lower than the values observed for the PEO-treated groups. Specifically, *R*_pin_ values for the PEO-treated samples were 5.15, 7.53, 6.44, and 11.1 GΩ·cm^2^ for CaP, Zn0.03, Zn0.05, and Zn0.1, respectively. By significantly amplifying *R*_pin_, these coatings act as effective barriers, minimizing corrosion process in the substrate interface. Capacitance (Q), modeled by constant phase elements, was lower for all PEO-treated groups compared to cpTi (*p* < 0.001; Figure 6f), with Zn0.1 showing the lowest capacitance, indicating a more stable and less reactive surface. Polarization resistance (Rp), reflecting corrosion resistance, was significantly higher in Zn0.1 than in other groups (Figure 6g). These properties suggest that Zn0.1 offers superior resistance to electrochemical degradation, reducing the risk of ion leaching and associated biological adverse reactions. Potentiodynamic polarization tests further highlighted the improved performance of PEO coatings against localized corrosion. Treated groups exhibited more positive polarization curves and higher corrosion potentials (*E*_corr_) at lower current densities than cpTi (*p* < 0.05; Figure 6h,i). Although the passivation current density (*i*_corr_) was similar among PEO-treated groups (*p* > 0.05), it was significantly lower than that of cpTi (*p* < 0.001; Figure 6j).

The anodic (βa) and cathodic (βc) Tafel constants are also critical parameters for evaluating the electrochemical performance of materials, as they reflect the kinetics of anodic and cathodic reactions during corrosion. In this study, the βa values for cpTi, CaP, Zn0.03, Zn0.05, and Zn0.1 were 2.04, 1.40, 1.82, 1.43, and 1.30 V/decade, respectively. Similarly, the βc values for these groups were 0.19, 2.22, 1.17, 1.51, and 0.63 V/decade, respectively. The cpTi exhibited the highest βa value, indicating slower anodic reaction kinetics. This suggests a higher activation barrier for anodic dissolution, which aligns with the lack of a protective coating. However, its extremely low βc value indicates fast cathodic kinetics, which can exacerbate corrosion in environments where reduction reactions dominate.^34^ In contrast, the PEO-treated groups demonstrated significantly lower βa values, particularly Zn0.1, suggesting improved anodic kinetics due to the formation of a stable oxide layer. The moderate βc values PEO-treated compared to cpTi samples suggests that the coatings reduce the cathodic reaction rate, thereby limiting the electron supply for the anodic reaction. This is beneficial as it slows down the corrosion cycle, providing greater electrochemical stability.

Corrosion rate analysis (Figure 6k) revealed that PEO-treated surfaces exhibited significantly lower corrosion rates compared to untreated cpTi (5.75 × 10^–3^ mpy, *p* < 0.001). Among the PEO-treated samples, the corrosion rates were as follows: CaP (3.14 × 10^–4^ mpy), Zn0.03 (1.86 × 10^–4^ mpy), Zn0.05 (1.81 × 10^–4^ mpy), and Zn0.1 (6.96 × 10^–6^ mpy). The substantial reduction in corrosion rates for the PEO-treated groups, particularly for the Zn0.1 coating, highlights its potential for enhancing implant longevity. This enhanced protection is especially relevant for implants subjected to highly corrosive conditions, such as in the oral environment.

The enhanced corrosion resistance observed in the Zn0.1 group is probably resulting from various factors involving the presence of titanium dioxide (TiO_2_) and zinc oxide (ZnO). The porous structure inherent to PEO coatings plays a nuanced role in determining their corrosion resistance. While porosity is often considered a vulnerability due to the increased likelihood of electrolyte penetration, the specific characteristics of the pores—such as size, distribution, and the crystallinity of the surrounding material—can profoundly influence this behavior.^21^ In the case of the Zn0.1 coating, despite having a higher pore count compared to other groups, its smaller pore diameters likely limited pathways for electrolyte diffusion, effectively reducing the risk of substrate corrosion. This balance between pore amount and diameter mitigates the typical vulnerabilities associated with porous structures, ensuring that the oxide layer retains its protective functionality even under highly corrosive conditions.

Furthermore, the presence of zinc oxide within the matrix contributes to sealing microstructural gaps, enhancing the compactness and stability of the coating.^56^ Notably, despite the greater porosity, Zn0.1 demonstrated superior performance, which can be attributed not only to its controlled pore architecture but also to the enhanced crystallinity of its pore walls. The XRD analysis revealed more intense diffraction peaks for Zn0.1, particularly for the rutile phase. Rutile is known for its higher structural density and reduced number of defects, resulting in a more regular atomic arrangement. By providing fewer interstitial spaces, the rutile phase forms a compact and chemically stable barrier, significantly improving the corrosion resistance of the Zn0.1 coating.

Moreover, zinc may provide a sacrificial protective effect. In standard conditions, zinc has an electrode potential (E^0^) of approximately −0.76 V in the Zn^2+^/Zn redox couple, while titanium standard electrode potential (Ti^4+^/Ti) is around −1.63 V.^54^ Despite titanium having a lower electrode potential in isolated conditions, in the context of corrosion in electrolytic environments, higher reactivity and superficial exposure probably lead zinc to be oxidize first. In practice, when both metals are exposed to an electrolyte, zinc will lose electrons more readily, undergoing oxidation and protecting the titanium surface by preventing its oxidation. This sacrificial protection mechanism, combined with the formation of a denser and uniform physical barrier, can significantly reduce the corrosion rate, extending the lifespan of the implant in corrosive environments, such as oral cavity. In a clinical context, the improved electrochemical stability of Zn0.1-coated implants could reduce the risk of corrosion-related complications, such as implant degradation and metal ion release, which are associated with inflammatory responses and peri-implant diseases.^8^

### 3.4 Reduced Microbial Colonization and Biofilm Viability on Titanium Surfaces

Rough implant surfaces often exhibit superior biological properties compared to polished, machined surfaces.^46^ However, increased roughness can also promote microbial colonization and biofilm formation, heightening the risk of peri-implant diseases. In this study, the zinc-incorporated coatings were evaluated for their ability to resist to the colonization by *S. sanguinis* and *E. coli*, model species representing dental and orthopedic implant infections, respectively.

**Figure 7:**
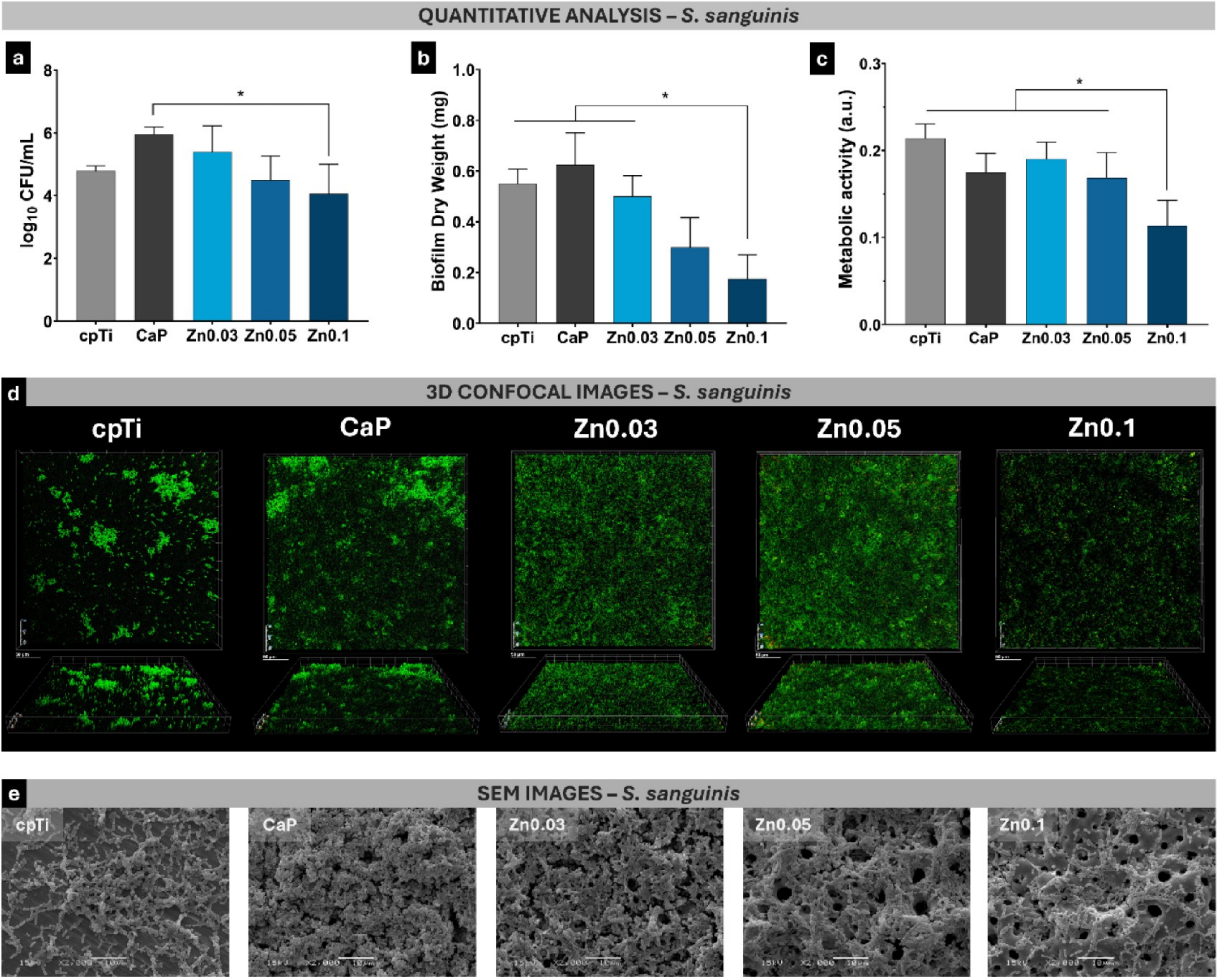
Antibacterial activity of control and zinc-containing experimental surfaces against *Streptococcus sanguinis* biofilm. (a) Colony-forming unit count (log_10_ CFU/mL) of *S. sanguinis* after 24 h of biofilm formation (*n* = 6). (b) Dry weight (mg) of *S. sanguinis* biofilm (*n* = 4). (c) Metabolic activity (a.u.) of *S. sanguinis* evaluated by XTT assay (absorbance values at 492 nm) (*n* = 4). (d) Two-dimensional (top) and three-dimensional (bottom) reconstructions of live/dead bacterial biofilm after 24 h of *S. sanguinis* biofilm formation (*n* = 1). Images were obtained using fluorescence staining (green for live bacteria, red for dead bacteria), with merged channels. Scale bars = 50 μm for X-Y surfaces. (e) SEM micrographs (*n* = 1) illustrating *S. sanguinis* colonization on surfaces (2000× magnification, 15 kV). Data are expressed as mean ± standard deviation. Statistically significant differences between groups are indicated by symbols, with * representing *p* < 0.05 in the Tukey HSD test.

*S. sanguinis* is a Gram-positive bacterium important in the initial stages of biofilm formation on dental implants.^41^ As a primary colonizer, it facilitates the coaggregation of other oral bacteria, promoting biofilm maturation and anaerobic conditions, being a key pathogen for dental implant-related infection.^14^ This process can lead to complex, pathogenic biofilms associated with peri-implant mucositis and peri-implantitis, triggering immune responses, inflammation, bone loss, and implant failure.^11^ Therefore, controlling the adhesion and biofilm formation of *S. sanguinis* is crucial in preventing the progression of peri-implant diseases, making it an important target for antimicrobial surface treatments. The microbiological assays demonstrated a significant antimicrobial effect of zinc-incorporated coatings compared to nonzinc coating. For *S. sanguinis*, the Zn0.1 group exhibited a statistically significant reduction in viable bacterial counts compared to the CaP group (*p* = 0.019; Figure 7a). This was further corroborated by the biofilm dry weight analysis, which showed reduction larger than 60% in biofilm biomass on the Zn0.1 surface compared to the CaP group (*p* < 0.0001) and all other groups (*p* < 0.002), except for Zn0.05 (Figure 7b). Additionally, XTT assays revealed reduced metabolic activity on the Zn0.1 surface compared to the other groups (*p* < 0.036; Figure 7c). CLSM images (Figures 7d and S4) further supported these findings, showing significantly lower viable cell biovolume on the Zn0.1 surface (1.99 ± 0.89 × 10^4^ μm^3^) compared to CaP (22.28 ± 0.39 × 10^4^ μm^3^). The Zn0.1 group also exhibited higher dead cell biovolume (3.94 ± 0.11 × 10^3^ μm^3^) compared to cpTi (0.57 ± 0.39 × 10^3^ μm^3^). Scanning electron micrographs (Figure 7e) confirmed these observations, showing extensive bacterial colonization on CaP, whereas Zn0.1 demonstrated reduced biofilm formation with fewer bacteria and less extracellular matrix formation, comparable to smooth cpTi.

**Figure 8:**
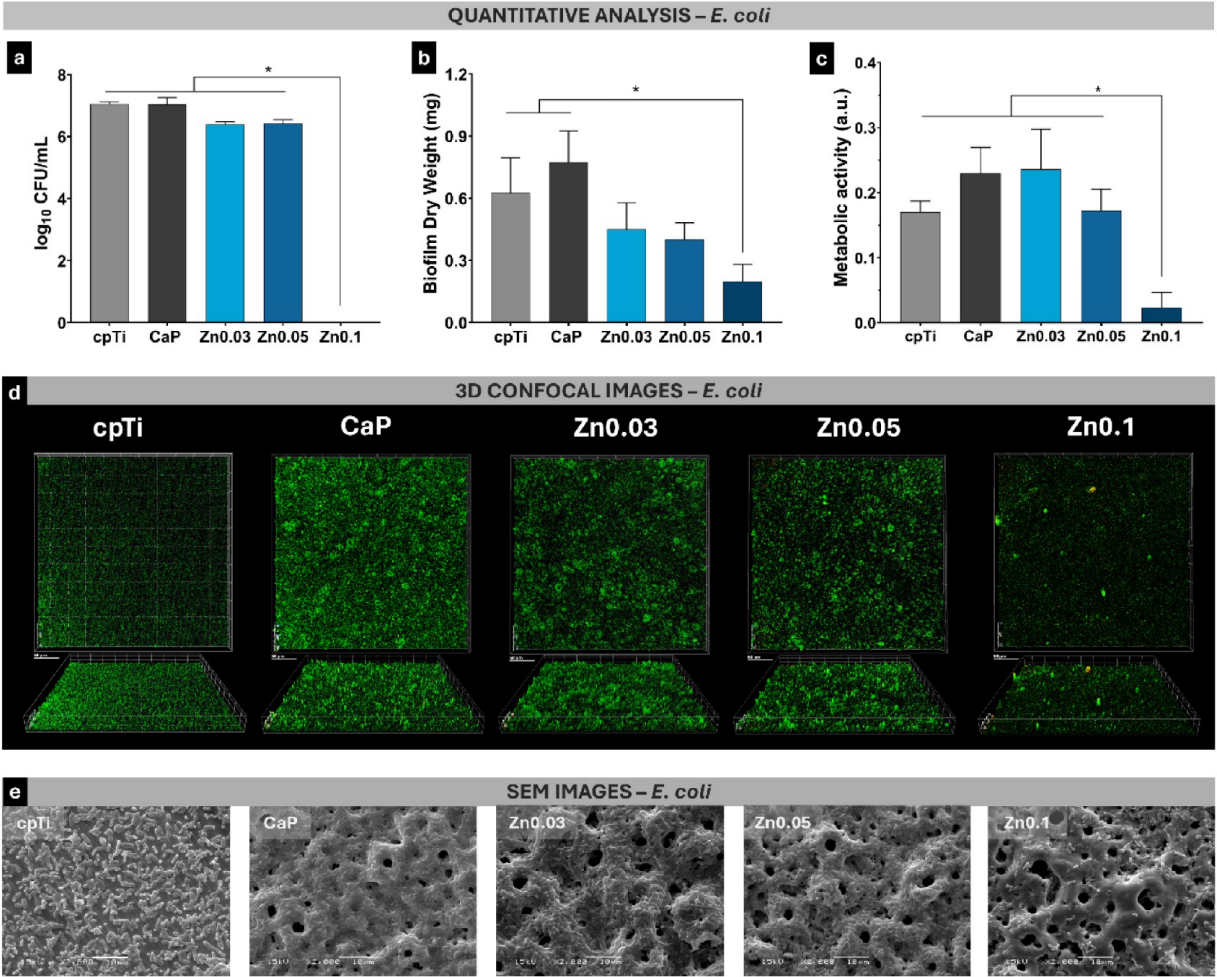
Antibacterial activity of control and zinc-containing experimental surfaces against *Escherichia coli* biofilm. (a) Colony-forming unit count (log_10_ CFU/mL) of *E. coli* after 24 h of biofilm formation. (b) Dry weight (mg) of *E. coli* biofilm (*n* = 4). (c) Metabolic activity (a.u.) of *E. coli* evaluated by XTT assay (absorbance values at 492 nm) (*n* = 4). (d) Two-dimensional (top) and three-dimensional (bottom) reconstructions of live/dead bacterial biofilm after 24 h of *E. coli* biofilm formation (*n* = 1). Images were obtained using fluorescence staining (green for live bacteria, red for dead bacteria), with merged channels. Scale bars = 50 μm for X-Y surfaces. (e) SEM photomicrographs (*n* = 1) illustrating *E. coli* colonization on surfaces (2000× magnification, 15 kV). Data are expressed as mean ± standard deviation. Statistically significant differences between groups are indicated by symbols, with * representing *p* < 0.05 in the Tukey HSD test.

In contrast, *E. coli* is a Gram-negative bacterium more commonly associated with orthopedic implant infections.^41^*E. coli* can cause deep-seated infections, especially in orthopedic implants, where it forms biofilms that can be resistant to conventional antibiotic therapies. Once biofilms are established, *E. coli* can evade the host immune response and persist on the implant surface, often leading to chronic infections that require implant removal.^41^ The ability to prevent *E. coli* colonization on implant surfaces is therefore critical for improving the success rate of orthopedic implant surgeries. Interestingly, zinc had a stronger antimicrobial effect on *E. coli* than on *S. sanguinis*. No viable *E. coli* cells were detected on the Zn0.1 surface, while the other zinc-coated groups also showed approximately a 1-log reduction in viable counts compared to the control groups (*p* < 0.001; Figure 8a). The Zn0.1 group exhibited significantly reduced biofilm formation, as indicated by a dry weight of approximately 0.2 mg, compared to ∼0.6 mg in the nonzinc groups (Figure 8b). Residual *E. coli* cells on Zn0.1 showed minimal metabolic activity (*p* < 0.001; Figure 8c), which may have contributed to the absence of viable cells in the CFU assay, which can be limited in certain circumstances. CLSM images (Figures 8d and S5) revealed an increased presence of dead cells, particularly in Zn0.1. Zn0.1 showed a significantly higher dead cell biovolume (12.58 ± 0.39 × 10^3^ μm^3^) compared to CaP (3.54 ± 1.01 × 10^3^ μm^3^) and cpTi (1.36 ± 0.08 × 10^3^ μm^3^). SEM micrographs (Figure 8e) confirmed these trends, with minimal biofilm matrix and reduced bacterial population observed on Zn0.1.

The differential antimicrobial efficacy of the zinc-incorporated coatings against *S. sanguinis* and *E. coli* observed in this study can likely be attributed to fundamental differences in the structural and physiological characteristics of these two bacteria. Gram-positive bacteria present a thick peptidoglycan layer in its cell wall that lacks an outer membrane, providing it with a rigid, yet less permeable, structure which may render *S. sanguinis* less susceptible to zinc ions. In contrast, *E. coli*, a Gram-negative bacterium, possesses an outer membrane composed of lipopolysaccharides (LPS) that may be more vulnerable to zinc ions.^41^ Different zinc forms, such as nanoparticles or ions, can disrupt the integrity of this outer membrane, leading to increased permeability, destabilization of membrane, and eventual cell death.^26^ The outer membrane of Gram-negative bacteria also contains porins, protein channels that regulate the passage of molecules, which may allow zinc ions easier access to intracellular targets.^57^ These combined factors explain why zinc exhibited a stronger effect on *E. coli* in terms of biofilm inhibition, metabolic reduction, and cell viability, while *S. sanguinis* remained relatively more resilient.

The antimicrobial properties of zinc are attributed to a multifaceted array of mechanisms that disrupt bacterial homeostasis, leading to cell death. One of the primary mechanisms is the interaction of zinc ions with bacterial cell membranes.^58,59^ This disruption increases membrane permeability, resulting in the leakage of essential cellular contents and the collapse of the membrane potential, which is critical for bacterial survival. Additionally, zinc ions can bind to sulfhydryl (−SH) groups in bacterial proteins, particularly enzymes, altering their conformation and inhibiting their function interfering with bacterial metabolism, replication and protein synthesis, essential biological process for biofilm extracellular matrix production.^60^ This disruption in microbial metabolism may explain the significantly reduced metabolic activity observed with the Zn0.1 coating, which in turn contributes to its notably lower biofilm formation, as evidenced by the decreased dry weight. Another important antibacterial mechanism of Zn is its ability to generate intracellular reactive oxygen species (ROS) that causes bacterial oxidative stress, leading to damage to cellular components such as lipids, proteins, and nucleic acids.^61^ Additionally, zinc has been shown to interfere with bacterial DNA replication by binding to zinc-finger domains in transcription factors or enzymes involved in the replication process, preventing proper DNA unwinding and replication.^26^

In clinical settings, it is understood that implant surfaces are constantly exposed to a variety of microorganisms, leading to inevitable bacterial colonization over time.^13^ While it is difficult to completely prevent bacterial attachment, the key challenge lies in addressing the formation of biofilms, which represent one of the most significant hurdles in managing peri-implant health.^14^ Moreover, biofilms create an environment that is particularly resistant to decontamination, making it more difficult for both patients and medical professionals to effectively clean the implant surfaces, whether through routine oral hygiene practices or specialized decontamination techniques.^17,42^ Additionally, biofilms serve as reservoirs for microbial cells, allowing bacteria to be released from the biofilm matrix and potentially colonize other sites in the body, further complicating patient outcomes.^11^ The ability of the Zn0.1 coating to significantly reduce biofilm formation is thus of great clinical importance. By minimizing biofilm development, the Zn0.1 coating can contribute to a more manageable implant environment, reducing the risk of infection spread and improving the overall efficacy of professional decontamination protocols and self-oral hygiene. This, in turn, could lead to fewer complications, longer implant survival, and better long-term patient outcomes in both dental and orthopedic applications.

### 3.5 Bioactivity and Cytocompatibility

**Figure 9:**
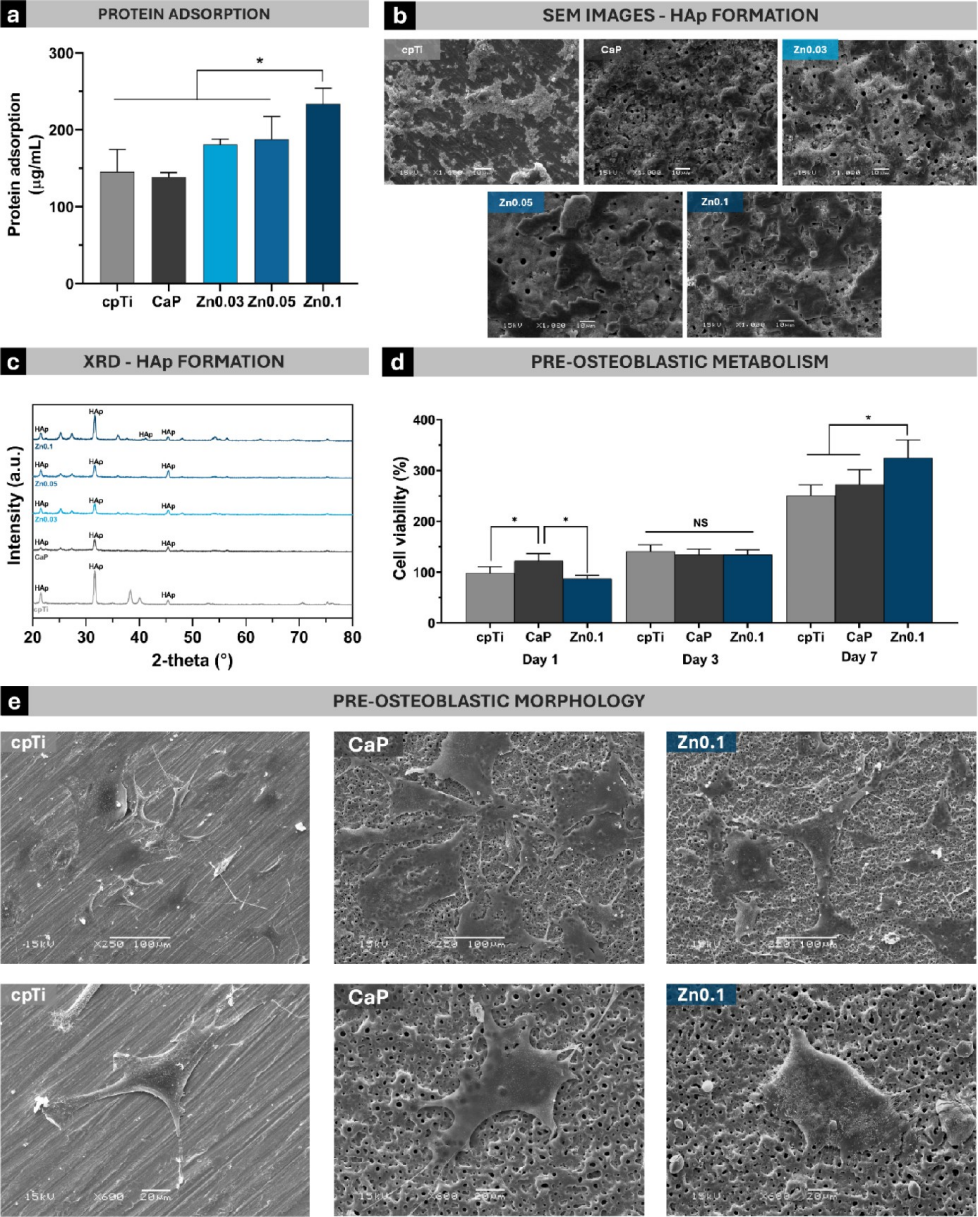
Protein adsorption, hydroxyapatite formation, and cellular response on control and PEO-treated surfaces. (a) Protein adsorption on control and PEO-treated surfaces (*n* = 3). Data are presented as mean ± standard deviation. (b) SEM micrographs and (c) X-ray diffraction pattern obtained after 28 days of immersion in SBF, showing the morphology and crystallinity of hydroxyapatite, respectively (*n* = 1). (d) Cell viability (*n* = 5) and (e) morphology (*n* = 2) of preosteoblastic MC3T3-E1 cells cultured on the control and experimental surfaces, evaluated by alamarBlue assay and SEM micrographs, respectively. Data are expressed as mean ± standard deviation, with * indicating statistically significant differences (*p* < 0.05) based on Tukey HSD test.

Coating’s bioactivity can be predicted by several factors, protein adsorption being one of them. In this study, human blood plasma was used to simulate protein pellicle formation on the implant surface during implantation. The results revealed that the addition of zinc in the coating significantly enhanced protein adsorption in a concentration-dependent manner. Notably, Zn0.1 group adsorbed approximately 220 μg/mL of protein, compared to less than 150 μg/mL in the control groups (*p* < 0.01; Figure 9a). Several factors, such as roughness and wettability, can contribute to protein adsorption.^35^ However, while all PEO-treated groups exhibited similar roughness (Ra) values and hydrophilic behavior, those factors alone do not account for the differences observed in protein adsorption across the groups. Therefore, the increased adsorption in the zinc-doped coatings, particularly Zn0.1, can probably be attributed to the presence of zinc itself. First, the increased formation of hydroxyl groups and rutile crystalline phase in these groups can contribute to an increased interaction between coating surface and negatively charged protein chains or amine groups, promoting stronger binding.^48^ Additionally, the presence of Zn^2+^ ions likely also enhanced electrostatic interactions between negatively charged proteins and the surface,^62^ also further increasing adsorption. Moreover, nearly 10% of the human proteome is known to bind to zinc, and the higher concentration of Zn^2+^ in the Zn0.1 coating could provide specific binding sites for proteins with zinc-binding domains.^30^ On implant surfaces, Zn^2+^ ions may enhance protein adsorption and stabilize zinc-binding proteins, which in turn could promote cellular pathways toward osseointegration.

Furthermore, the bioactivity of both control and zinc-incorporated experimental surfaces was also evaluated based on their ability to precipitate calcium phosphate. All surfaces demonstrated the capacity to induce hydroxyapatite formation (HAp) and growth, confirming their potential to support bone integration. However, significant differences were observed in the morphology of the HAp layer between the surface types, as shown in the micrographs (Figure 9b). While the cpTi surface exhibited a classical granular HAp morphology, likely due to its polished nature, the PEO-treated groups—especially those containing zinc—showed a distinctive plate-like quadrangular structure. This finding aligns with a previous study that reported similar morphological patterns in HAp formation on rougher and more irregular surfaces which may provide more nucleation sites for the deposition of bioactive ceramic layers.^35^ Yet, the increased hydrophilicity of the coatings may have contributed to better precipitation of HAp in the surface.^34^ This is particularly promising for the Zn0.1 group, as the formation of a bioactive ceramic layer is critical for facilitating direct bonding with bone tissue and promoting osseointegration.^34^ Additionally, XRD analysis (Figure 9c) confirmed that the structures formed on the sample surfaces were indeed calcium phosphate crystals (HAp peaks at 2theta ∼31°).^35^ Notably, a distinct peak appeared exclusively in the Zn0.1 group (HAp peak at 2theta ∼40°), suggesting the formation of a more crystalline and potentially bioactive HAp layer.^35^ The EDS analysis (Figure S6) revealed that the products formed after 28 days of immersion in SBF were primarily composed of Ca, P, and O, suggesting the presence of calcium oxide and phosphate, as well as a significant amount of calcium phosphate (Ca–P) compounds. Notably, in the zinc-doped groups, the Ca/P ratio approached approximately 1.4, which is close to the stoichiometric ratio of hydroxyapatite.^35^ These findings are further supported by the XRD analysis, which confirmed that the immersion products are consistent with the formation of hydroxyapatite (HA), a biologically relevant calcium phosphate phase. The presence of hydroxyapatite on the coating surface can significantly influence cell behavior by providing a favorable substrate for cell adhesion, proliferation, and differentiation.^34^ The combination of controlled zinc release with the formation of HA-like deposits suggests that these coatings may promote an osteoconductive environment, further supporting their potential in biomedical applications.

In this sense, the viability of preosteoblastic MC3T3-E1 cells cultured on control surfaces (cpTi and CaP) and the experimental Zn0.1 surface was also assessed over 1, 3, and 7 days. On day 1, cells cultured on the CaP surface exhibited the highest metabolic activity (*p* < 0.0013; Figure 9d), even surpassing cpTi, which served as the 100% reference. While Zn0.1 did not promote the same initial cell activity as CaP, probably due to the more rapid Zn ion release within 24 h, it maintained cell viability above 70%, which is considered the threshold for cytotoxicity according to ISO 10993-5. The increased metabolic activity observed on the CaP surface on day 1 could potentially be attributed to its surface properties. One hypothesis is that the CaP coating might offer a larger surface area for initial cell attachment, which could enable faster adhesion and higher early metabolic rates. By day 3, no significant differences in metabolic activity were detected between the three tested surfaces (*p* > 0.05; Figure 9d), indicating comparable cell viability across all groups. However, on day 7, cells cultured on the Zn0.1 surface displayed significantly higher metabolic activity compared to both CaP and cpTi (*p* ≤ 0.005; Figure 9d), suggesting a delayed but enhanced cellular response. The improvement in cell viability and morphology observed on the Zn0.1 surface may be linked to the sustained release profile of Zn ions from the coating even after a week, which could potentially contribute to cellular activity. Zn has been shown to play an essential role in intracellular processes that promote osteoblast differentiation and function.^50^

Yet, the role of cell-material interaction was further demonstrated by SEM micrographs (Figure 9e), showing differences in cell morphology after 24 h of culture. In the cpTi group, cells sparsely covered the surface, and at higher magnification, they appeared more rounded, with limited spreading and only a few cytoplasmic extensions. These extensions are critical for adhesion, yet their reduced presence suggests a weaker interaction with the surface. This behavior is likely due to the bioinert nature of titanium, which, despite its cytocompatibility, does not actively promote bioactivity. In contrast, CaP and Zn0.1 groups exhibited cells with well-organized cytoskeletal structures and an increased number of filopodial extensions. At higher magnifications, cells displayed well-defined morphologies with extended pseudopodia anchored to surface valleys and peaks and interconnected with the porous structures, indicating strong cell attachment.^63^ These findings suggest that the porous topography of PEO-treated surfaces plays a key role in guiding cell morphology and adhesion. The combination of surface structure and chemical composition appears to enhance cell attachment, spreading, and organization, ultimately promoting a more favorable cellular response on PEO-treated surfaces compared to untreated titanium, which could positively affect secondary implant stability and clinical outcomes.

### 3.6 Improved Resistance and Implant Stability *Ex Vivo*

**Figure 10:**
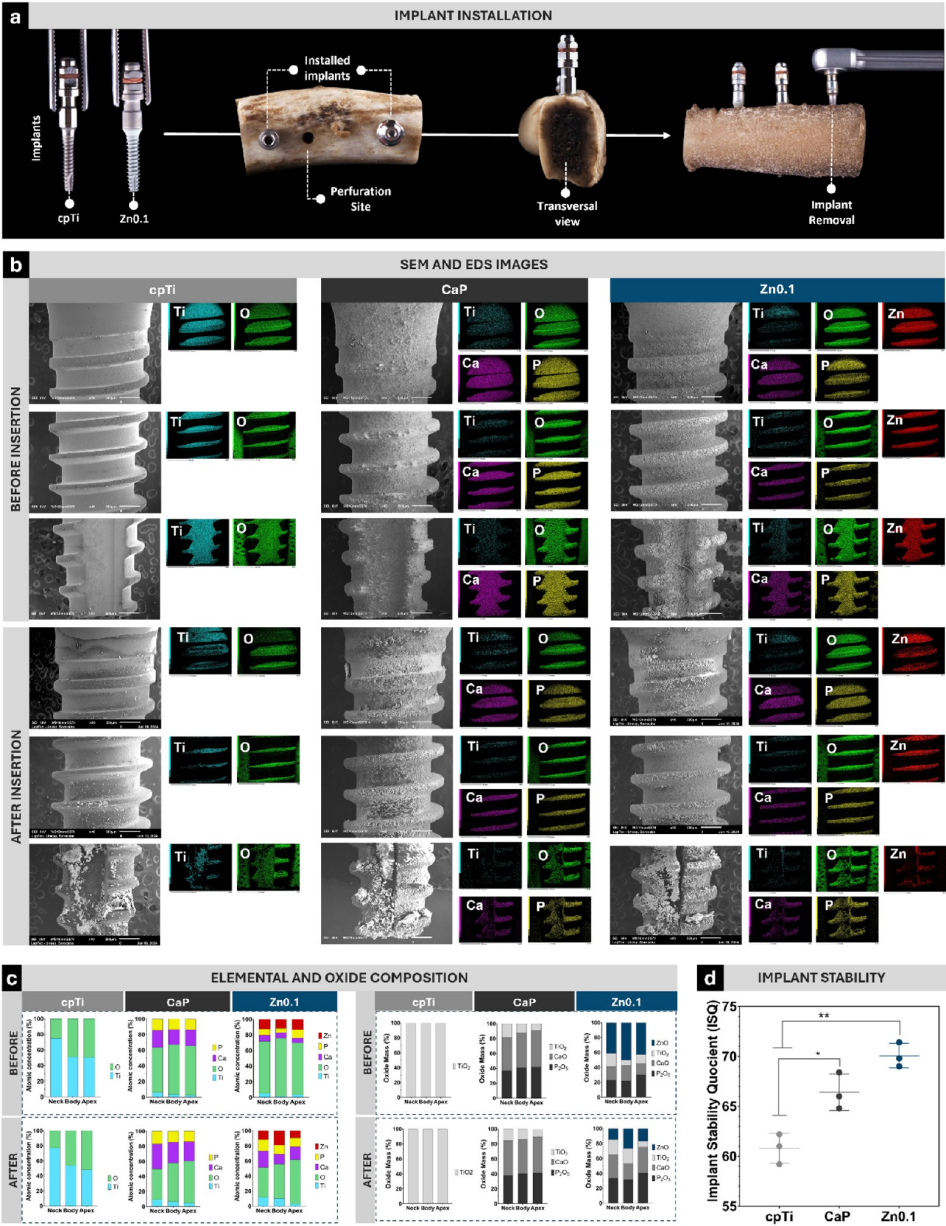
*Ex vivo* characterization of control and PEO-treated implants after insertion into bovine bone. (a) Macroscopic comparison of implant surfaces between the smooth cpTi group and Zn0.1. The drilling site is shown in the top view and transverse bone view demonstrates implant positioning within the bone. The final image illustrates the process of implant removal using a ratchet after insertion. (b) SEM micrographs of implant surfaces before and after insertion into bone, showing the smooth surface of cpTi and the porous morphology of CaP and Zn0.1 groups (40× magnification, 8 kV). (c) EDS atomic percentage composition of before and after coatings insertion, demonstrating the elemental distribution, with emphasis on the preservation of zinc content and overall coating integrity. (d) Implant Stability Quotient (ISQ) values measured for each group after insertion, indicating increased stability for PEO-treated implants, particularly Zn0.1 (*n* = 5). Data are expressed as mean ± standard deviation. Statistically significant differences between groups are indicated by symbols, with * representing *p* < 0.05 in the Tukey HSD test.

First, it is important to note that differently from other surface modification methods, the PEO treatment was successfully applied to titanium disks and implants, resulting in the formation of microstructured surfaces with distinct macroscopic characteristics (Figure 10a). SEM micrographs of the implants before insertion revealed the smooth surface of the cpTi group, while both the CaP and Zn0.1 groups exhibited a characteristic porous morphology (Figure 10b). Notably, the Zn0.1 group displayed a more homogeneous and well-distributed porous structure, indicating the formation of a consistent coating in terms of morphology compared to SEM micrographs of *in vitro* analysis in Ti disks. The EDS chemical composition analysis confirmed the presence of zinc oxide in the Zn0.1 group, showing a stable incorporation of zinc within the oxide layer (Figure 10c). These findings demonstrate the reliability and reproducibility of the PEO process in creating bioactive and microstructured surfaces, even on the complex geometry of dental implants. Following implant insertion and removal from the bone site, SEM showed that the PEO coatings remained largely intact, with no significant morphological alterations or delamination observed, which corroborates with the wear track characterization in the *in vitro* tests. Even in the helical grooves—areas typically more susceptible to mechanical damage during insertion^7^—there was no evidence of coating damage.

Furthermore, EDS color maps and oxide proportion composition analysis indicated no substantial loss of zinc from the coating postinsertion, similarly to what have been seen in the tribological results, further underscoring the robustness of the PEO-generated oxide layer. These *ex vivo* results align with the wear resistance observed *in vitro*, where the Zn0.1 coating also exhibited minimal degradation under mechanical load. The high resistance of the implants with Zn0.1 coating to insertional damage is also likely attributed to the formation of the rutile phase of titanium dioxide in the oxide structure.^38^ The rutile phase is known for its higher microhardness,^37^ which may contribute to the increased durability of the coating during implant placement. This is a crucial advantage, as previous studies have shown that other surface treatments can promote particles release to the bone site upon insertion.^7,64^ Furthermore, the increased resistance to mechanical degradation observed in PEO-coatings suggests that they could minimize wear and particle release during implant placement, reducing the likelihood of associated inflammatory complications.

In addition to the preservation of morphological features, the PEO-treated coatings, particularly Zn0.1, demonstrated a marked improvement in implant stability, as evidenced by increased implant stability quotient (ISQ) values (*p* < 0.01; Figure 10d). Implant stability is traditionally categorized into two distinct phases: primary and secondary stability. Primary stability refers to the mechanical engagement of the implant with the bone at the time of insertion, driven by factors such as bone quality, implant design, and surface characteristics.^65^ In this study, the enhanced primary stability of the PEO-treated implants can be attributed to the increased surface area and porous topography.^66^ The creation of a porous surface may enhance interlocking between the implant and the bone, thereby facilitating bone ingrowth. Such interlocking, facilitated by enhanced roughness, may contribute to a higher implant stability reducing the chances of micromotions, thereby mitigating the risk of fibrous tissue formation between the implant and the bone.^67^

This greater interlocking effect was further confirmed by SEM micrographs after implant removal, revealing residual bone tissue still adhered to the CaP and Zn0.1 coatings, providing direct evidence of improved surface interaction. This improved initial stability plays a pivotal role in supporting the healing process, as it reduces the risk of early micromovements that could compromise osseointegration.^44^ Also, as healing progresses, primary stability transitions into secondary stability, which is achieved through osseointegration,^50,54^ a complex biological process that could be benefited by zinc coatings considering its bioactivity verified in this study.

Therefore, the enhanced implant stability provided by PEO-treated surfaces could be particularly advantageous in reducing the likelihood of deleterious micromotions during the critical early stages of healing. This is especially relevant in clinical scenarios involving poor-quality bone or in patients with systemic conditions that impair bone quality and osseointegration^18^ both of which complicate immediate prosthetic rehabilitation and jeopardize long-term implant success. Additionally, the improved stability of Zn0.1-coated implants may allow for immediate prosthetic loading, reducing the need for extended healing periods and allowing for earlier functionality, enhancing both patient outcomes and procedural efficiency.

### 3.7 Practical Implications and Future Research Directions

The findings of this study highlight the significant clinical and industrial potential of simplified zinc-doped PEO coatings for titanium implants. By addressing critical challenges such as mechanical durability, electrochemical stability, biofilm inhibition, and bioactivity, these coatings, particularly Zn0.1, offer a highly promising solution for enhancing implant performance in both dental and orthopedic applications. Clinically, the Zn0.1 coating’s superior mechanical resistance, high corrosion stability, and antimicrobial properties provide a practical advantage in preventing biofilm-associated infections and ensuring long-term implant functionality. Its ability to resist insertional damage and promote osseointegration makes it particularly valuable in high-risk scenarios, such as patients with diabetes or osteoporosis, where healing is impaired, and infection risks are heightened. The zinc ion release profile further supports its role in combating early stage infections while facilitating bone healing.

Nonetheless, some limitations warrant further investigation. First, the *in vitro* model may not fully capture the complexities of the clinical environment, where factors like mechanical loading, immune responses, and diverse microbial challenges could affect implant performance. Therefore, future *in vivo* and clinical studies are essential to fully elucidate the impact of zinc-doped coatings under more dynamic and biologically challenging conditions. Furthermore, the molecular mechanisms by which zinc exerts its antibacterial effects and promotes bioactivity remain unclear, underscoring the need for further research to elucidate these pathways. While this study confirmed enhanced protein adsorption on Zn0.1 coatings, a more in-depth exploration of the proteomic profile of the acquired pellicle is needed. This could provide valuable insights into the specific proteins involved and their roles in facilitating osseointegration.

## 4 Conclusions

This study successfully produced zinc-doped PEO coatings on titanium implants using a single plasma electrolytic oxidation approach, effectively integrating enhanced mechanical and corrosion resistance, antibacterial properties, and bioactivity into a unified system. The incorporation of zinc into the oxide layer proved to be a key factor in improving the multifunctional performance of the coatings. Among the tested groups, Zn0.1 demonstrated superior outcomes, exhibiting enhanced mechanical and corrosion resistance, hydroxyapatite formation and protein adsorption. The Zn-doped coatings also showed promising antibacterial activity, with Zn0.1 reducing *S. sanguinis* and *E. coli* biofilm’s viability, metabolism and biomass. Additionally, cytocompatibility assays confirmed increased preosteoblastic cell metabolism and morphological features supporting their potential for osseointegration. This study provides a streamlined approach for fabricating bioactive coatings that combine corrosion resistance, antibacterial properties, and bioactivity. The results highlight the potential of zinc-doped PEO coatings for challenging clinical applications, such as dental and orthopedic implants, offering enhanced performance in demanding biological environments.

## Supporting Information Available

High-resolution confocal laser scanning microscopy (CLSM) images at 50× magnification merged with optical images; the complete X-ray photoelectron spectroscopy (XPS) full spectrum of the Zn0.1 group detailing chemical composition and bonding states; scanning electron microscopy (SEM) micrographs of PEO-treated surfaces following the coating adhesion test, CLSM images including separate live (green) and dead (red) bacterial channels for *Streptococcus sanguinis* and *Escherichia coli*; and energy-dispersive X-ray spectroscopy (EDS) maps demonstrating elemental presence after 28 days of immersion in simulated body fluid (SBF) (PDF)

## References

[ref1] Pavel K.; Seydlova M.; Dostalova T.; Zdenek V.; Chleborad K.; Jana Z.; Feberova J.; Radek H. Dental Implants and Improvement of Oral Health-Related Quality of Life. Community Dent. Oral. Epidemiol. 2012, 40, 65–70. 10.1111/j.1600-0528.2011.00668.x. 22369711

[ref2] De Avila E. D.; Nagay B. E.; Pereira M. M. A.; Barão V. A. R.; Pavarina A. C.; Van Den Beucken J. J. J. P. Race for Applicable Antimicrobial Dental Implant Surfaces to Fight Biofilm-Related Disease: Advancing in Laboratorial Studies vs Stagnation in Clinical Application. ACS Biomater. Sci. Eng. 2022, 8 (8), 3187–3198. 10.1021/acsbiomaterials.2c00160. 35816289

[ref3] Cordeiro J. M.; Barão V. A. R. Is There Scientific Evidence Favoring the Substitution of Commercially Pure Titanium with Titanium Alloys for the Manufacture of Dental Implants?. Mater. Sci. Eng. 2017, 71, 1201–1215. 10.1016/j.msec.2016.10.025. 27987677

[ref4] Frisch E.; Wild V.; Ratka-Krüger P.; Vach K.; Sennhenn-Kirchner S. Long-Term Results of Implants and Implant-Supported Prostheses under Systematic Supportive Implant Therapy: A Retrospective 25-Year Study. Clin. Implant Dent. Relat. Res. 2020, 22 (6), 689–696. 10.1111/cid.12944. 32969180

[ref5] Nagay B. E.; Cordeiro J. M.; Barao V. A. R. Insight Into Corrosion of Dental Implants: From Biochemical Mechanisms to Designing Corrosion-Resistant Materials. Curr. Oral Health Rep. 2022, 9 (2), 7–21. 10.1007/s40496-022-00306-z. 35127334 PMC8799988

[ref6] Atalay P. Mechanical Complications of Dental Implants: A Review. Open Access J. Dent. Sci. 2022, 7 (4), 156–158. 10.23880/oajds-16000349.

[ref7] Senna P.; Antoninha Del Bel Cury A.; Kates S.; Meirelles L. Surface Damage on Dental Implants with Release of Loose Particles after Insertion into Bone. Clin. Implant Dent. Relat. Res. 2015, 17 (4), 681–692. 10.1111/cid.12167. 24283455 PMC4420732

[ref8] Noronha Oliveira M.; Schunemann W. V. H.; Mathew M. T.; Henriques B.; Magini R. S.; Teughels W.; Souza J. C. M. Can. Degradation Products Released from Dental Implants Affect Peri-Implant Tissues?. J. Periodontal Res. 2018, 53, 1–11. 10.1111/jre.12479. 28766712

[ref9] Chen L.; Tong Z.; Luo H.; Qu Y.; Gu X.; Si M. Titanium Particles in Peri-Implantitis: Distribution, Pathogenesis and Prospects. Int. J. Oral Sci. 2023, 15, 49 10.1038/s41368-023-00256-x. 37996420 PMC10667540

[ref10] Souza J. G. S.; Costa Oliveira B. E.; Bertolini M.; Lima C. V.; Retamal-Valdes B.; de Faveri M.; Feres M.; Barão V. A. R. Titanium Particles and Ions Favor Dysbiosis in Oral Biofilms. J. Periodontal Res. 2020, 55 (2), 258–266. 10.1111/jre.12711. 31762055

[ref11] Schwarz F.; Derks J.; Monje A.; Wang H. L. Peri-Implantitis. J. Clin. Periodontol. 2018, 45, S246–S266. 10.1111/jcpe.12954. 29926484

[ref12] Sgolastra F.; Petrucci A.; Severino M.; Gatto R.; Monaco A. Periodontitis, Implant Loss and Peri-Implantitis: A Meta-Analysis. Clin Oral Implants Res. 2015, 26 (4), e8–e16. 10.1111/clr.12319. 24382358

[ref13] Belibasakis G. N.; Manoil D. Microbial Community-Driven Etiopathogenesis of Peri-Implantitis. J. Dent. Res. 2021, 100, 21–28. 10.1177/0022034520949851. 32783779 PMC7754824

[ref14] Kotsakis G. A.; Olmedo D. G. Peri-Implantitis Is Not Periodontitis: Scientific Discoveries Shed Light on Microbiome-Biomaterial Interactions That May Determine Disease Phenotype. Periodontol. 2000 2021, 86 (1), 231–240. 10.1111/prd.12372. 33690947

[ref15] Flemming H. C.; van Hullebusch E. D.; Neu T. R.; Nielsen P. H.; Seviour T.; Stoodley P.; Wingender J.; Wuertz S. The Biofilm Matrix: Multitasking in a Shared Space. Nat. Rev. Microbiol. 2023, 21, 70–86. 10.1038/s41579-022-00791-0. 36127518

[ref16] Romandini M.; Lima C.; Pedrinaci I.; Araoz A.; Soldini M. C.; Sanz M. Prevalence and Risk/Protective Indicators of Peri-Implant Diseases: A University-Representative Cross-Sectional Study. Clin Oral Implants Res. 2021, 32 (1), 112–122. 10.1111/clr.13684. 33210772

[ref17] Ramanauskaite A.; Fretwurst T.; Schwarz F. Efficacy of Alternative or Adjunctive Measures to Conventional Non-Surgical and Surgical Treatment of Peri-Implant Mucositis and Peri-Implantitis: A Systematic Review and Meta-Analysis. Int. J. Implant Dent. 2021, 7 (1), 112 10.1186/s40729-021-00388-x. 34779939 PMC8593130

[ref18] Mosaddad S. A.; Talebi S.; Keyhan S. O.; Fallahi H. R.; Darvishi M.; Aghili S. S.; Tavahodi N.; Namanloo R. A.; Heboyan A.; Fathi A. Dental Implant Considerations in Patients with Systemic Diseases: An Updated Comprehensive Review. J. Oral Rehabil. 2024, 51, 1250–1302. 10.1111/joor.13683. 38570927

[ref19] Parihar A.; Madhuri S.; Devanna R.; Sharma G.; Singh R.; Shetty K. Assessment of Failure Rate of Dental Implants in Medically Compromised Patients. J. Fam. Med. Prim Care 2020, 9 (2), 883 10.4103/jfmpc.jfmpc_989_19. PMC7113960 32318439

[ref20] Malheiros S. S.; Nagay B. E.; Bertolini M. M.; de Avila E. D.; Shibli J. A.; Souza J. G. S.; Barão V. A. R. Biomaterial Engineering Surface to Control Polymicrobial Dental Implant-Related Infections: Focusing on Disease Modulating Factors and Coatings Development. Expert Rev. Med. Devices 2023, 20 (7), 557–573. 10.1080/17434440.2023.2218547. 37228179

[ref21] Costa R. C.; Nagay B. E.; Dini C.; Borges M. H. R.; Miranda L. F. B.; Cordeiro J. M.; Souza J. G. S.; Sukotjo C.; Cruz N. C.; Barão V. A. R. The Race for the Optimal Antimicrobial Surface: Perspectives and Challenges Related to Plasma Electrolytic Oxidation Coating for Titanium-Based Implants. Adv. Colloid Interface Sci. 2023, 311, 102805 10.1016/j.cis.2022.102805. 36434916

[ref22] van Hengel I. A. J.; Tierolf M. W. A. M.; Fratila-Apachitei L. E.; Apachitei I.; Zadpoor A. A. Antibacterial Titanium Implants Biofunctionalized by Plasma Electrolytic Oxidation with Silver, Zinc, and Copper: A Systematic Review. Int. J. Mol. Sci. 2021, 22, 3800 10.3390/ijms22073800. 33917615 PMC8038786

[ref23] Sopchenski L.; Popat K.; Soares P. Bactericidal Activity and Cytotoxicity of a Zinc Doped PEO Titanium Coating. Thin Solid Films 2018, 660, 477–483. 10.1016/j.tsf.2018.05.055.

[ref24] Zuo K.; Wang L.; Wang Z.; Yin Y.; Du C.; Liu B.; Sun L.; Li X.; Xiao G.; Lu Y. Zinc-Doping Induces Evolution of Biocompatible Strontium–Calcium-Phosphate Conversion Coating on Titanium to Improve Antibacterial Property. ACS Appl. Mater. Interfaces 2022, 14 (6), 7690–7705. 10.1021/acsami.1c23631. 35114085

[ref25] Jiang S.; Lin K.; Cai M. ZnO Nanomaterials: Current Advancements in Antibacterial Mechanisms and Applications. Front. Chem. 2020, 8, 580 10.3389/fchem.2020.00580. 32793554 PMC7385224

[ref26] Vitasovic T.; Caniglia G.; Eghtesadi N.; Ceccato M.; Bo̷jesen E. D.; Gosewinkel U.; Neusser G.; Rupp U.; Walther P.; Kranz C.; et al. Antibacterial Action of Zn2+ Ions Driven by the In Vivo Formed ZnO Nanoparticles. ACS Appl. Mater. Interfaces 2024, 16 (24), 30847–30859. 10.1021/acsami.4c04682. 38853353

[ref27] Wen Z.; Shi X.; Li X.; Liu W.; Liu Y.; Zhang R.; Yu Y.; Su J. Mesoporous TiO2 Coatings Regulate ZnO Nanoparticle Loading and Zn 2+ Release on Titanium Dental Implants for Sustained Osteogenic and Antibacterial Activity. ACS Appl. Mater. Interfaces 2023, 15 (12), 15235–15249. 10.1021/acsami.3c00812. 36926829

[ref28] Hu H.; Zhang W.; Qiao Y.; Jiang X.; Liu X.; Ding C. Antibacterial Activity and Increased Bone Marrow Stem Cell Functions of Zn-Incorporated TiO2 Coatings on Titanium. Acta Biomater. 2012, 8 (2), 904–915. 10.1016/j.actbio.2011.09.031. 22023752

[ref29] Santos-Coquillat A.; Mohedano M.; Martinez-Campos E.; Arrabal R.; Pardo A.; Matykina E. Bioactive Multi-Elemental PEO-Coatings on Titanium for Dental Implant Applications. Mater. Sci. Eng. 2019, 97, 738–752. 10.1016/j.msec.2018.12.097. 30678963

[ref30] Coverdale J. P. C.; Barnett J. P.; Adamu A. H.; Griffiths E. J.; Stewart A. J.; Blindauer C. A. A Metalloproteomic Analysis of Interactions between Plasma Proteins and Zinc: Elevated Fatty Acid Levels Affect Zinc Distribution. Metallomics 2019, 11 (11), 1805–1819. 10.1039/C9MT00177H. 31612889

[ref31] Zhao B. H.; Zhang W.; Wang D. N.; Feng W.; Liu Y.; Lin Z.; Du K. Q.; Deng C. F. Effect of Zn Content on Cytoactivity and Bacteriostasis of Micro-Arc Oxidation Coatings on Pure Titanium. Surf. Coat. Technol. 2013, 228, S428–S432. 10.1016/j.surfcoat.2012.05.037.

[ref32] Du Q.; Wei D.; Wang Y.; Cheng S.; Liu S.; Zhou Y.; Jia D. The Effect of Applied Voltages on the Structure, Apatite-Inducing Ability and Antibacterial Ability of Micro Arc Oxidation Coating Formed on Titanium Surface. Bioact. Mater. 2018, 3 (4), 426–433. 10.1016/j.bioactmat.2018.06.001. 29988748 PMC6031222

[ref33] Leśniak-Ziółkowska K.; Kazek-Kęsik A.; Rokosz K.; Raaen S.; Stolarczyk A.; Krok-Borkowicz M.; Pamuła E.; Gołda-Cępa M.; Brzychczy-Włoch M.; Simka W. Electrochemical Modification of the Ti-15Mo Alloy Surface in Solutions Containing ZnO and Zn3(PO4)2 Particles. Mater. Sci. Eng. 2020, 115, 111098 10.1016/j.msec.2020.111098. 32600702

[ref34] Borges M. H. R.; Nagay B. E.; Costa R. C.; Sacramento C. M.; Ruiz K. G.; Landers R.; van den Beucken J. J. J. P.; Fortulan C. A.; Rangel E. C.; da Cruz N. C.; et al. A Tattoo-Inspired Electrosynthesized Polypyrrole Film: Crossing the Line toward a Highly Adherent Film for Biomedical Implant Applications. Mater. Today Chem. 2022, 26, 101095 10.1016/j.mtchem.2022.101095.

[ref35] Costa R. C.; Souza J. G. S.; Cordeiro J. M.; Bertolini M.; de Avila E. D.; Landers R.; Rangel E. C.; Fortulan C. A.; Retamal-Valdes B.; da Cruz N. C.; Feres M.; Barão V. A. R. Synthesis of Bioactive Glass-Based Coating by Plasma Electrolytic Oxidation: Untangling a New Deposition Pathway toward Titanium Implant Surfaces. J. Colloid Interface Sci. 2020, 579, 680–698. 10.1016/j.jcis.2020.06.102. 32652323

[ref36] Cordeiro J. M.; Nagay B. E.; Dini C.; Souza J. G. S.; Rangel E. C.; da Cruz N. C.; Yang F.; van den Beucken J. J. J. P.; Barão V. A. R. Copper Source Determines Chemistry and Topography of Implant Coatings to Optimally Couple Cellular Responses and Antibacterial Activity. Biomater. Adv. 2022, 134, 112550 10.1016/j.msec.2021.112550. 35523647

[ref37] Silva J. P. D. S.; Costa R. C.; Nagay B. E.; Borges M. H. R.; Sacramento C. M.; da Cruz N. C.; Rangel E. C.; Fortulan C. A.; da Silva J. H. D.; Ruiz K. G. S.; et al. Boosting Titanium Surfaces with Positive Charges: Newly Developed Cationic Coating Combines Anticorrosive and Bactericidal Properties for Implant Application. ACS Biomater. Sci. Eng. 2023, 9 (9), 5389–5404. 10.1021/acsbiomaterials.3c00491. 37561763

[ref38] Andrade C. S.; Borges M. H. R.; Silva J. P.; Malheiros S.; Sacramento C.; Ruiz K. G. S.; da Cruz N. C.; Rangel E. C.; Fortulan C.; Figueiredo L.; et al. Micro-Arc Driven Porous ZrO2 Coating for Tailoring Surface Properties of Titanium for Dental Implants Application. Colloids Surf., B 2025, 245, 114237 10.1016/j.colsurfb.2024.114237. 39293292

[ref39] Dini C.; Costa R. C.; Bertolini M.; Shibli J. A.; Feres M.; Klein M. I.; de Avila É. D.; Souza J. G. S.; Barão V. A. R. In-Vitro Polymicrobial Oral Biofilm Model Represents Clinical Microbial Profile and Disease Progression during Implant-Related Infections. J. Appl. Microbiol. 2023, 134 (11), lxad265 10.1093/jambio/lxad265. 37951291

[ref40] Souza J. G. S.; Bertolini M.; Costa R. C.; Cordeiro J. M.; Nagay B. E.; de Almeida A. B.; Retamal-Valdes B.; Nociti F. H.; Feres M.; Rangel E. C.; Barão V. A. R. Targeting Pathogenic Biofilms: Newly Developed Superhydrophobic Coating Favors a Host-Compatible Microbial Profile on the Titanium Surface. ACS Appl. Mater. Interfaces 2020, 12 (9), 10118–10129. 10.1021/acsami.9b22741. 32049483

[ref41] Dini C.; Borges M. H. R.; Malheiros S. S.; Piazza R. D.; van den Beucken J. J. J. P.; de Avila E. D.; Souza J. G. S.; Barão V. A. R. Progress in Designing Therapeutic Antimicrobial Hydrogels Targeting Implant-associated Infections: Paving the Way for a Sustainable Platform Applied to Biomedical Devices. Adv. Healthcare Mater. 2025, 14 (2), 2402926 10.1002/adhm.202402926. 39440583

[ref42] Costa R. C.; Takeda T. T. S.; Dini C.; Bertolini M.; Ferreira R. C.; Pereira G.; Sacramento C. M.; Ruiz K. G. S.; Feres M.; Shibli J. A.; et al. Efficacy of a Novel Three-Step Decontamination Protocol for Titanium-Based Dental Implants: An in Vitro and in Vivo Study. Clin Oral Implants Res. 2024, 35 (3), 268–281. 10.1111/clr.14224. 38131526

[ref43] Nagay B. E.; Dini C.; Cordeiro J. M.; Ricomini-Filho A. P.; De Avila E. D.; Rangel E. C.; Da Cruz N. C.; Barão V. A. R. Visible-Light-Induced Photocatalytic and Antibacterial Activity of TiO2 Codoped with Nitrogen and Bismuth: New Perspectives to Control Implant-Biofilm-Related Diseases. ACS Appl. Mater. Interfaces 2019, 11 (20), 18186–18202. 10.1021/acsami.9b03311. 31038914

[ref44] Ivanova V.; Chenchev I.; Zlatev S.; Mijiritsky E. Correlation between Primary, Secondary Stability, Bone Density, Percentage of Vital Bone Formation and Implant Size. Int. J. Environ. Res. Public Health 2021, 18 (13), 6994 10.3390/ijerph18136994. 34208849 PMC8297224

[ref45] H H.; G W.; E H. The Clinical Significance of Implant Stability Quotient (ISQ) Measurements: A Literature Review. J. Oral Biol. Craniofacial Res. 2020, 10, 629–638. 10.1016/j.jobcr.2020.07.004. PMC7494467 32983857

[ref46] Zhang Y.; Chen S. E.; Shao J.; Van Den Beucken J. J. J. P. Combinatorial Surface Roughness Effects on Osteoclastogenesis and Osteogenesis. ACS Appl. Mater. Interfaces 2018, 10 (43), 36652–36663. 10.1021/acsami.8b10992. 30270615 PMC6213029

[ref47] Fröjd V.; Wennerberg A.; Franke Stenport V. Importance of Ca ^2+^ Modifications for Osseointegration of Smooth and Moderately Rough Anodized Titanium Implants – A Removal Torque and Histological Evaluation in Rabbit. Clin. Implant Dent. Relat. Res. 2012, 14 (5), 737–745. 10.1111/j.1708-8208.2010.00315.x. 20977616

[ref48] Barberi J.; Spriano S. Titanium and Protein Adsorption: An Overview of Mechanisms and Effects of Surface Features. Materials 2021, 14, 1590 10.3390/ma14071590. 33805137 PMC8037091

[ref49] Eriksson C.; Nygren H.; Ohlson K. Implantation of Hydrophilic and Hydrophobic Titanium Discs in Rat Tibia: Cellular Reactions on the Surfaces during the First 3 Weeks in Bone. Biomaterials 2004, 25 (19), 4759–4766. 10.1016/j.biomaterials.2003.12.006. 15120522

[ref50] He J.; Feng W.; Zhao B.-H.; Zhang W.; Lin Z. In Vivo Effect of Titanium Implants with Porous Zinc-Containing Coatings Prepared by Plasma Electrolytic Oxidation Method on Osseointegration in Rabbits. Int. J. Oral Maxillofac. Implants 2018, 33 (2), 298–310. 10.11607/jomi.5764. 29420672

[ref51] Zhang X.; Li C.; Yu Y.; Lu X.; Lv Y.; Jiang D.; Peng Z.; Zhou J.; Zhang X.; Sun S.; Dong Z. Characterization and Property of Bifunctional Zn-Incorporated TiO2Micro-Arc Oxidation Coatings: The Influence of Different Zn Sources. Ceram. Int. 2019, 45 (16), 19747–19756. 10.1016/j.ceramint.2019.06.228.

[ref52] Qiao L. P.; Lou J.; Zhang S. F.; Qu B.; Chang W. H.; Zhang R. F. The Entrance Mechanism of Calcium and Phosphorus Elements into Micro Arc Oxidation Coatings Developed on Ti6Al4V Alloy. Surf. Coat. Technol. 2016, 285, 187–196. 10.1016/j.surfcoat.2015.11.041.

[ref53] Stojadinović S.; Tadić N.; Vasilić R. Formation and Characterization of ZnO Films on Zinc Substrate by Plasma Electrolytic Oxidation. Surf. Coat. Technol. 2016, 307, 650–657. 10.1016/j.surfcoat.2016.09.080.

[ref54] Bordbar-Khiabani A.; Ebrahimi S.; Yarmand B. In-Vitro Corrosion and Bioactivity Behavior of Tailored Calcium Phosphate-Containing Zinc Oxide Coating Prepared by Plasma Electrolytic Oxidation. Corros. Sci. 2020, 173, 108781 10.1016/j.corsci.2020.108781.

[ref55] Cai Q.; Gao Y.; Gao T.; Lan S.; Simalou O.; Zhou X.; Zhang Y.; Harnoode C.; Gao G.; Dong A. Insight into Biological Effects of Zinc Oxide Nanoflowers on Bacteria: Why Morphology Matters. ACS Appl. Mater. Interfaces 2016, 8 (16), 10109–10120. 10.1021/acsami.5b11573. 27042940

[ref56] Bakhsheshi-Rad H. R.; Hamzah E.; Ismail A. F.; Aziz M.; Daroonparvar M.; Saebnoori E.; Chami A. In Vitro Degradation Behavior, Antibacterial Activity and Cytotoxicity of TiO2-MAO/ZnHA Composite Coating on Mg Alloy for Orthopedic Implants. Surf. Coat. Technol. 2018, 334, 450–460. 10.1016/j.surfcoat.2017.11.027.

[ref57] Mikhaylina A.; Ksibe A. Z.; Scanlan D. J.; Blindauer C. A. Bacterial Zinc Uptake Regulator Proteins and Their Regulons. Biochem. Soc. Trans. 2018, 46, 983–1001. 10.1042/BST20170228. 30065104 PMC6103462

[ref58] Stanić V.; Dimitrijević S.; Antić-Stanković J.; Mitrić M.; Jokić B.; Plećaš I. B.; Raičević S. Synthesis, Characterization and Antimicrobial Activity of Copper and Zinc-Doped Hydroxyapatite Nanopowders. Appl. Surf. Sci. 2010, 256 (20), 6083–6089. 10.1016/j.apsusc.2010.03.124.

[ref59] Wang Y.-W.; Cao A.; Jiang Y.; Zhang X.; Liu J.-H.; Liu Y.; Wang H. Superior Antibacterial Activity of Zinc Oxide/Graphene Oxide Composites Originating from High Zinc Concentration Localized around Bacteria. ACS Appl. Mater. Interfaces 2014, 6 (4), 2791–2798. 10.1021/am4053317. 24495147

[ref60] Moretti A. I. S.; Baksheeva V. E.; Roman A. Y.; De Bessa T. C.; Devred F.; Kovacic H.; Tsvetkov P. O. Exploring the Influence of Zinc Ions on the Conformational Stability and Activity of Protein Disulfide Isomerase. Int. J. Mol. Sci. 2024, 25 (4), 2095 10.3390/ijms25042095. 38396772 PMC10889200

[ref61] Appierot G.; Lipovsky A.; Dror R.; Perkas N.; Nitzan Y.; Lubart R.; Gedanken A. Enhanced Antibacterial Actiwity of Nanocrystalline ZnO Due to Increased ROS-Mediated Cell Injury. Adv. Funct. Mater. 2009, 19 (6), 842–852. 10.1002/adfm.200801081.

[ref62] Ren N.; Li J.; Qiu J.; Sang Y.; Jiang H.; Boughton R. I.; Huang L.; Huang W.; Liu H. Nanostructured Titanate with Different Metal Ions on the Surface of Metallic Titanium: A Facile Approach for Regulation of RBMSCs Fate on Titanium Implants. Small 2014, 10 (15), 3169–3180. 10.1002/smll.201303391. 24706634

[ref63] Matsuura T.; Komatsu K.; Cheng J.; Park G.; Ogawa T. Beyond Microroughness: Novel Approaches to Navigate Osteoblast Activity on Implant Surfaces. Int. J. Implant Dent. 2024, 10 (1), 35 10.1186/s40729-024-00554-x. 38967690 PMC11226592

[ref64] Franchi M.; Bacchelli B.; Martini D.; De Pasquale V.; Orsini E.; Ottani V.; Fini M.; Giavaresi G.; Giardino R.; Ruggeri A. Early Detachment of Titanium Particles from Various Different Surfaces of Endosseous Dental Implants. Biomaterials 2004, 25 (12), 2239–2246. 10.1016/j.biomaterials.2003.09.017. 14741589

[ref65] Monje A.; Ravidà A.; Wang H.-L.; Helms J.; Brunski J. Relationship Between Primary/Mechanical and Secondary/Biological Implant Stability. Int. J. Oral Maxillofac. Implants 2019, 34, s7–s23. 10.11607/jomi.19suppl.g1. 31116830 PMC13561118

[ref66] Romero M.; Herrero-Climent M.; Ríos-Carrasco B.; Brizuela A.; Romero M. M.; Gil J. Investigation of the Influence of Roughness and Dental Implant Design on Primary Stability via Analysis of Insertion Torque and Implant Stability Quotient: An In Vitro Study. J. Clin. Med. 2023, 12 (13), 4190 10.3390/jcm12134190. 37445228 PMC10342708

[ref67] Wazen R. M.; Currey J. A.; Guo H.; Brunski J. B.; Helms J. A.; Nanci A. Micromotion-Induced Strain Fields Influence Early Stages of Repair at Bone-Implant Interfaces. Acta Biomater. 2013, 9 (5), 6663–6674. 10.1016/j.actbio.2013.01.014. 23337705 PMC3622828

