# Supplementary material for: Zinc-Doped Antibacterial Coating as a Single Approach to Unlock Multifunctional and Highly Resistant Titanium Implant Surfaces
Source: ACS Appl Mater Interfaces. 2025 Mar 18;17(12):18022–45. doi: 10.1021/acsami.4c21875 (PMC11955950; doi:10.1021/acsami.4c21875)
Supplement: Supplementary file 1 — am4c21875_si_001.pdf [file am4c21875_si_001.pdf]

Supporting Information

Zinc-doped antibacterial coating as a single approach to unlock multifunctional and highly resistant titanium implant surfaces

Samuel S. Malheiros[a], Maria Helena R. Borges[a], Elidiane C. Rangel[b], Carlos A Fortulan[c], Nilson C. da Cruz[b], Valentim A. R. Barão[a], Bruna E. Nagay[a]**

a Department of Prosthodontics and Periodontology, Piracicaba Dental School, Universidade Estadual de Campinas (UNICAMP), Av. Limeira, 901, Piracicaba, São Paulo 13414-903, Brazil.

b Laboratory of Technological Plasmas, Institute of Science and Technology, São Paulo State University (UNESP), Av. Três de Março, 511, Sorocaba, São Paulo 18087-180, Brazil.

c Department of Mechanical Engineering, University of São Paulo (USP), Trabalhador São Carlense, 400, São Carlos, São Paulo 13566-590, Brazil.

***Corresponding author:**

Email address: bruna.eguminagay@hotmail.com (B. Nagay)

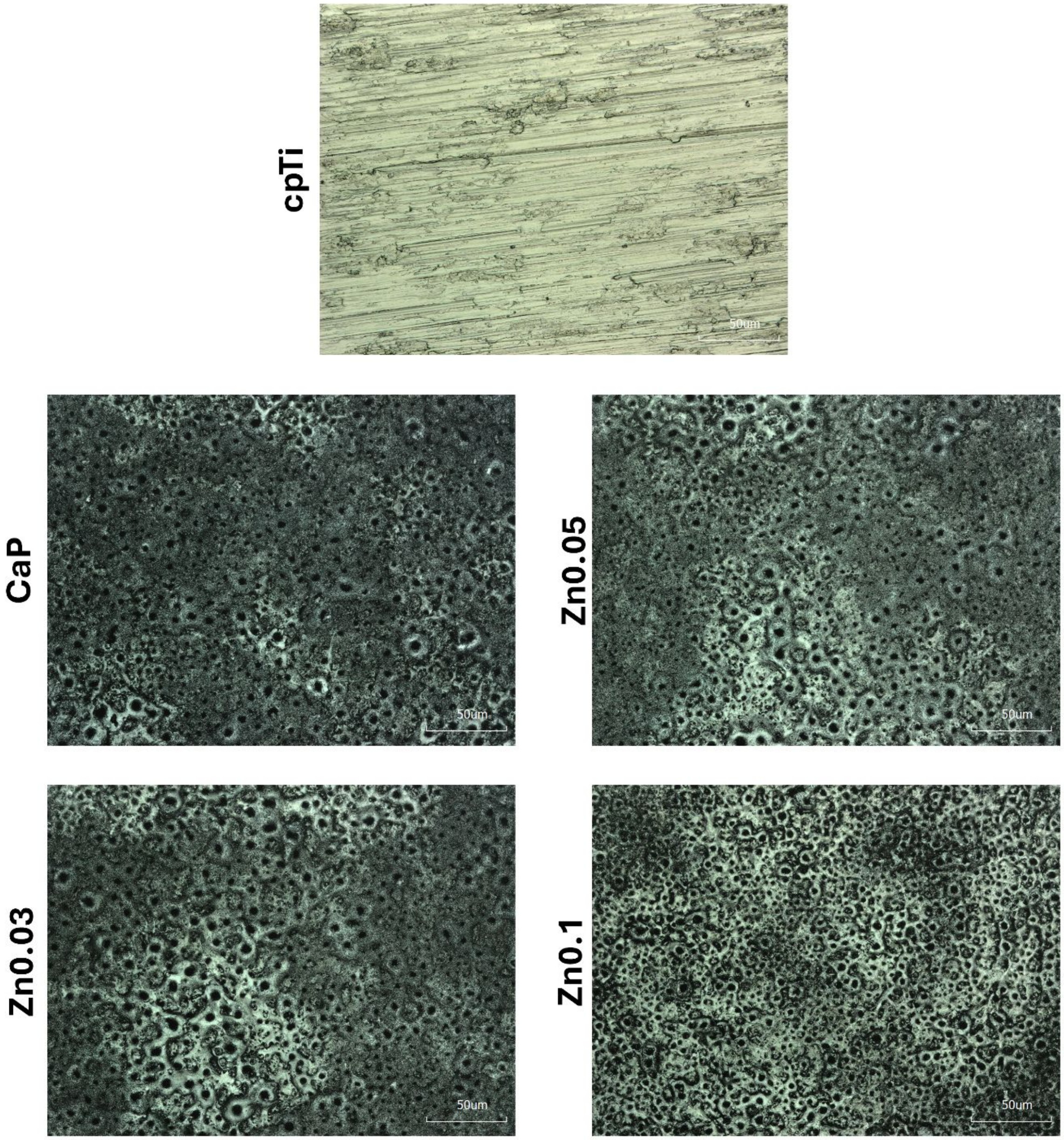

Figure S1. Confocal laser scanning microscopy (CLSM) images merged with optical images of sample surfaces. The merged visualization highlights the surface topography.

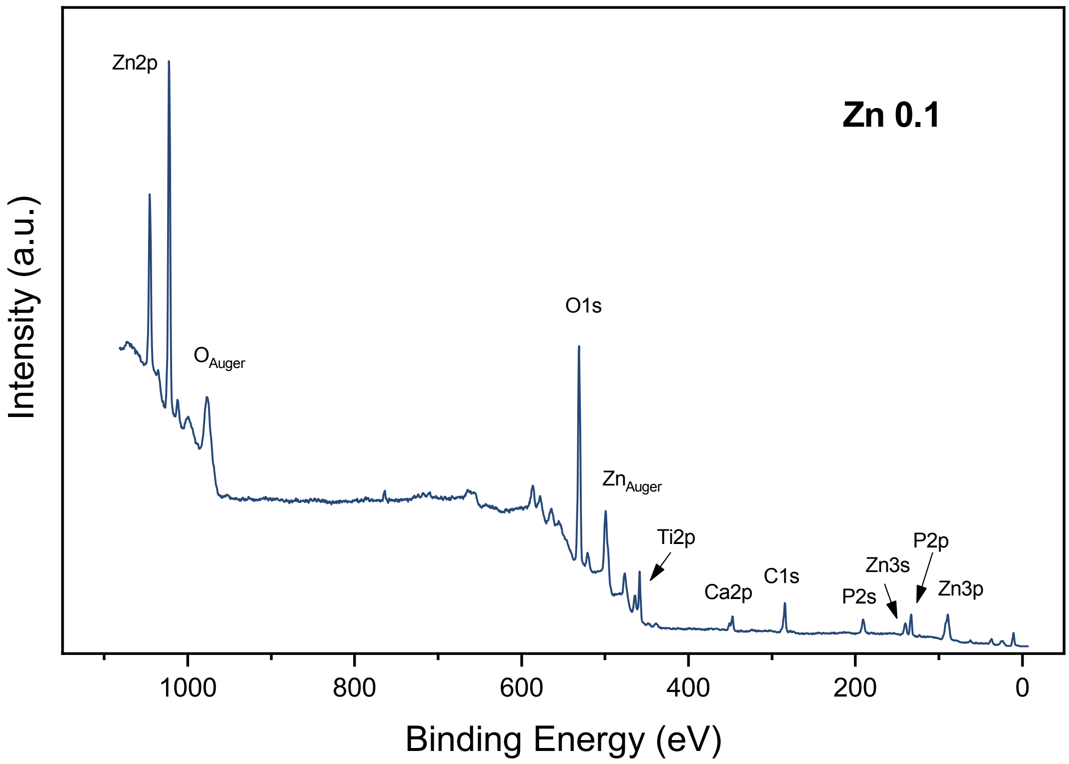

Figure S2. Full X-ray photoelectron spectroscopy (XPS) spectrum of the Zn0.1 group.

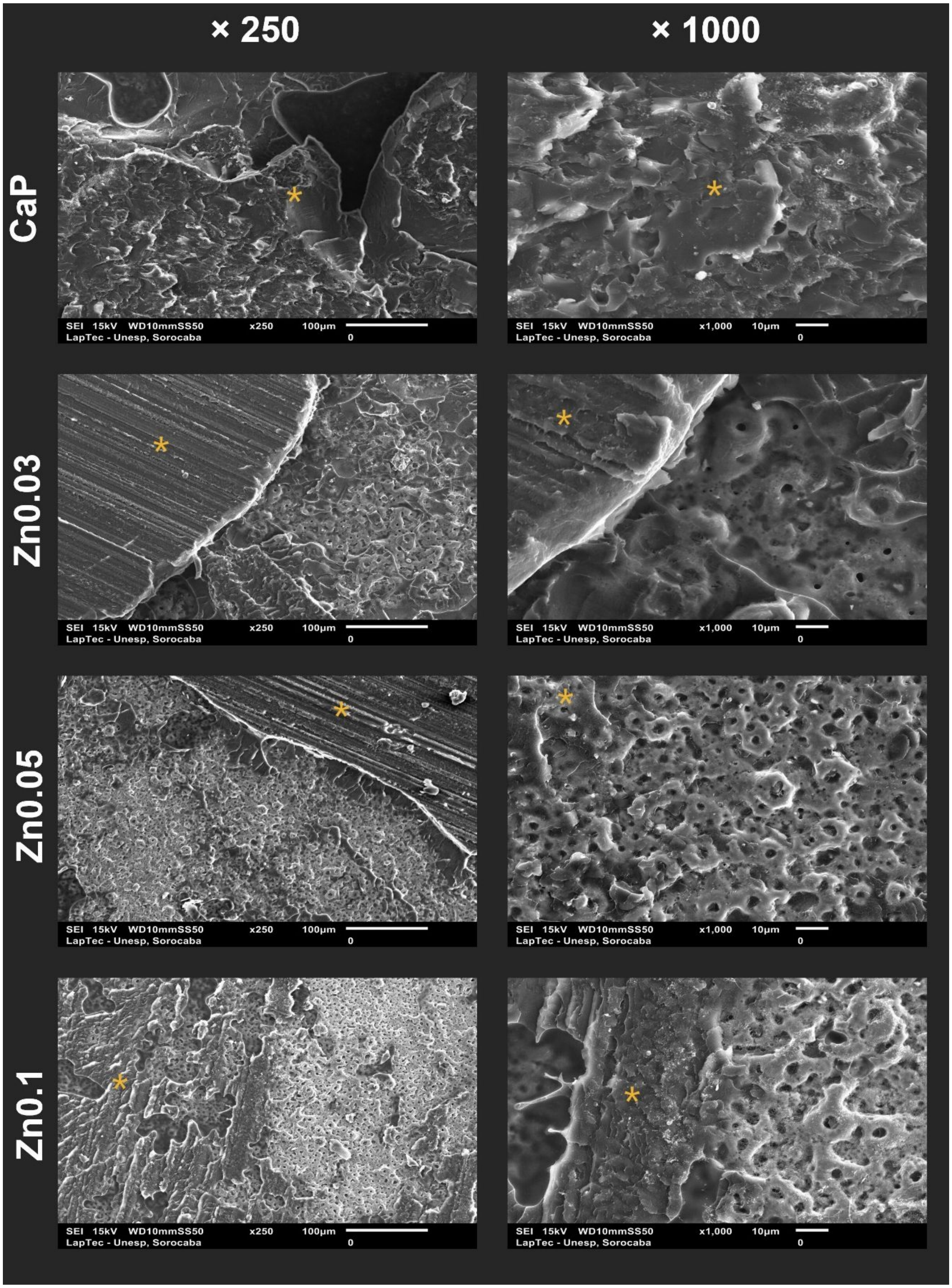

Figure S3. SEM micrographs of morphological features of failure sites from adhesion test. Yellow asterisks represent areas of cyanoacrylate still attached to coatings.

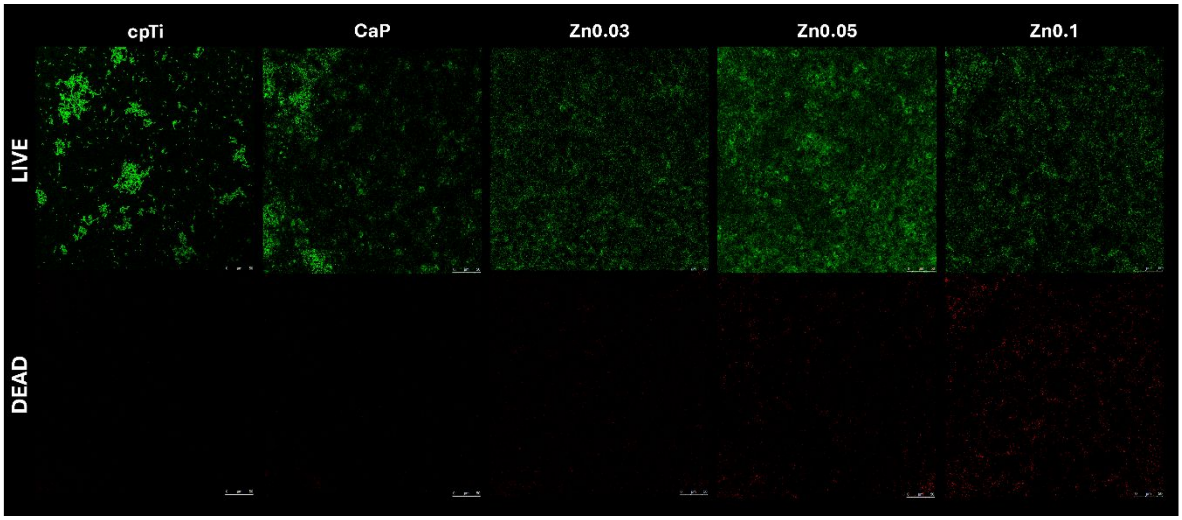

Figure S4. Two-dimensional projection of *Streptococcus sanguinis* biofilm after 24 hours of growth on Zn-doped PEO-coated surfaces. The image illustrates the spatial distribution of live (green) and dead (red) bacteria within the biofilm.

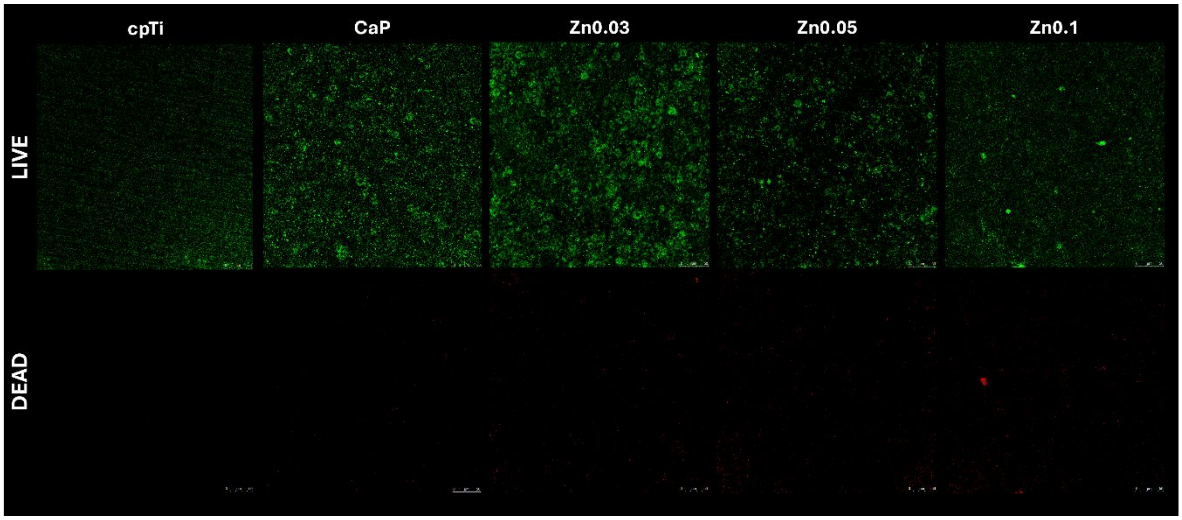

Figure S5. Two-dimensional projection of *Streptococcus sanguinis* biofilm after 24 hours of growth on Zn-doped PEO-coated surfaces. The image illustrates the spatial distribution of live (green) and dead (red) bacteria within the biofilm.

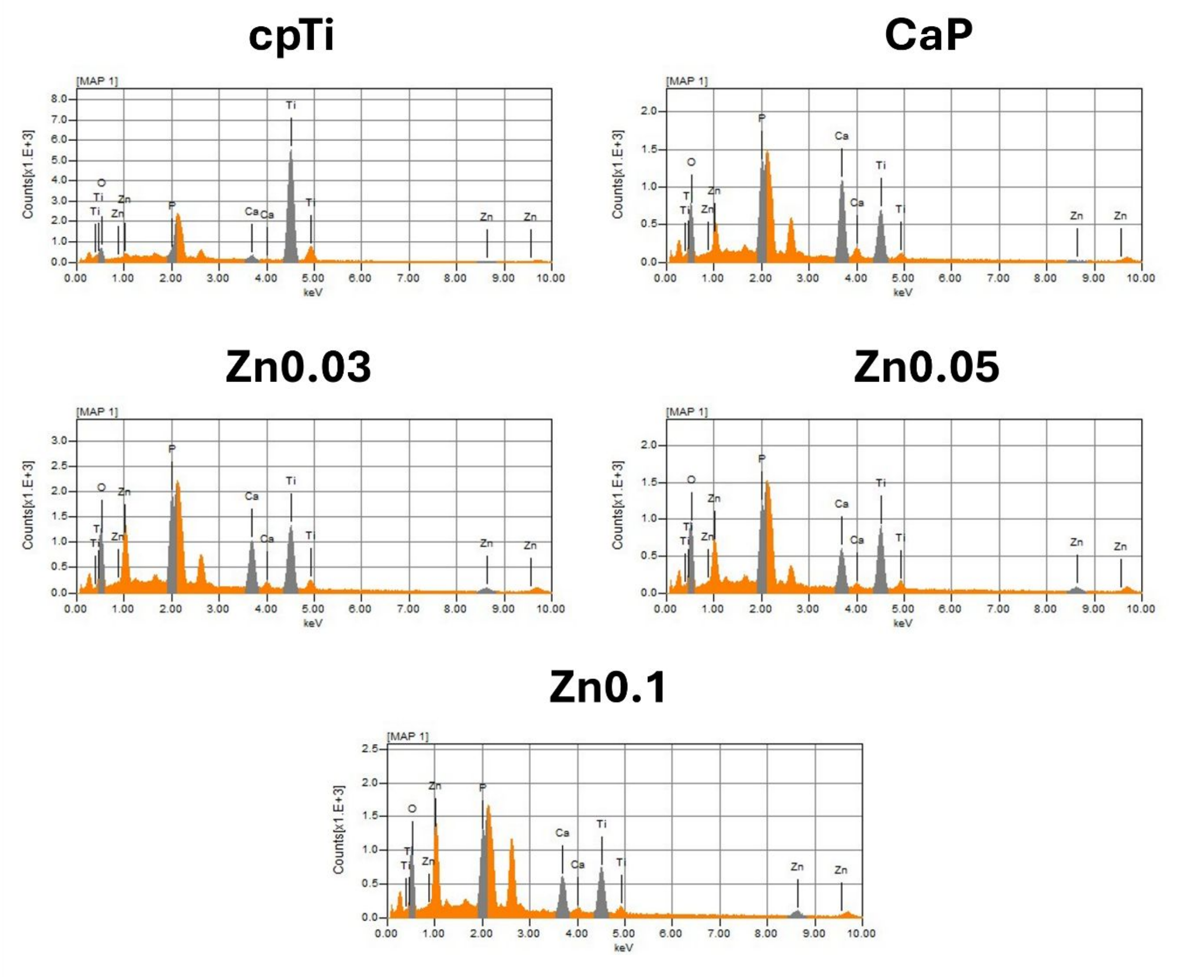

Figure S6. EDS analysis displaying the elemental composition of the surfaces after 28 days of immersion in SBF, with corresponding count maps as a function of energy (keV).
